# *klf2a* couples mechanotransduction and zebrafish valve morphogenesis through fibronectin synthesis

**DOI:** 10.1038/ncomms11646

**Published:** 2016-05-25

**Authors:** Emily Steed, Nathalie Faggianelli, Stéphane Roth, Caroline Ramspacher, Jean-Paul Concordet, Julien Vermot

**Affiliations:** 1 Institut de Génétique et de Biologie Moléculaire et Cellulaire, Illkirch 67404, France; 2 Centre National de la Recherche Scientifique, UMR7104, Illkirch 67404, France; 3 Institut National de la Santé et de la Recherche Médicale, U964, Illkirch 67404, France; 4 Université de Strasbourg, Illkirch 67404, France; 5 Muséum National d'Histoire Naturelle, 75231 Paris Cedex 05, France; 6 CNRS UMR 7196, 75231 Paris Cedex 05, France; 7 INSERM U1154, 75231 Paris Cedex 05, France

## Abstract

The heartbeat and blood flow signal to endocardial cell progenitors through mechanosensitive proteins that modulate the genetic program controlling heart valve morphogenesis. To date, the mechanism by which mechanical forces coordinate tissue morphogenesis is poorly understood. Here we use high-resolution imaging to uncover the coordinated cell behaviours leading to heart valve formation. We find that heart valves originate from progenitors located in the ventricle and atrium that generate the valve leaflets through a coordinated set of endocardial tissue movements. Gene profiling analyses and live imaging reveal that this reorganization is dependent on extracellular matrix proteins, in particular on the expression of *fibronectin1b*. We show that blood flow and *klf2a*, a major endocardial flow-responsive gene, control these cell behaviours and *fibronectin1b* synthesis. Our results uncover a unique multicellular layering process leading to leaflet formation and demonstrate that endocardial mechanotransduction and valve morphogenesis are coupled via cellular rearrangements mediated by fibronectin synthesis.

## Abstract (web summary)

In the developing heart, blood flow transmits mechanical signals to progenitor cells that ultimately leads to valve formation. Here, the authors identify the origin of the valve progenitor cells and *fibronectin1b* as a transcriptional target of the mechanotransduced signals responsible for valve formation.

## Graphical abstract

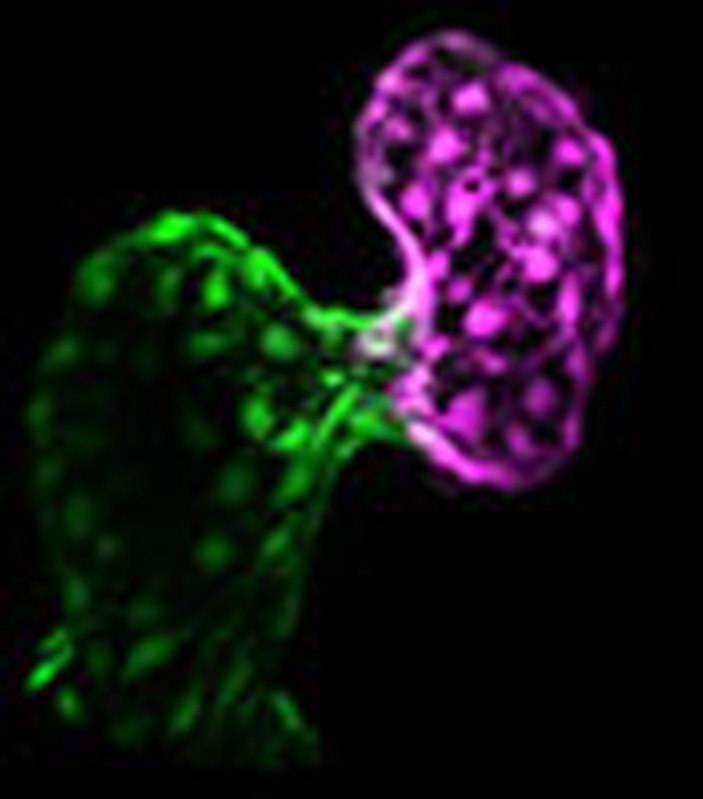

Tissue morphogenesis and organ formation depend upon the cooperative behaviour of groups of cells as well as the integration of chemical inputs generated in growing tissues. In addition, cells experience environmental mechanical stresses, such as pressure, strain and shear stress, due to tissue deformation and biological flows^1^,^2^, which subsequently participate in driving morphogenetic movements^1^,^3^,^4^,^5^. Due to the early initiation of heart contraction, the formation of the cardiovascular system is intricately linked to its function. Indeed, flow forces are necessary for cardiac ballooning, trabeculation and epicardium formation with flow forces being necessary for cardiac ballooning^6^, trabeculation^7^,^8^ and epicardium formation^9^. In both the lymphatic and cardiac systems, valves serve to maintain unidirectional fluid flow and, pertinently, depend on their respective flows to form^10^,^11^.

Congenital heart valve malformations constitute an important medical issue challenging our society. In recent years, it has become clear that most valve disease has its origin during embryogenesis, either as signs of abnormal developmental processes or the aberrant re-expression of fetal gene programs normally quiescent in adulthood^12^,^13^. These include mutations in genes encoding signalling factors (Notch1 and TGFβ)^14^ for the aortic valves, and actin-binding proteins (Filamin A)^15^ for the mitral valves. Diseased valves often also display defects in extracellular matrix (ECM) deposition^16^, which plays an essential function in valve architecture^17^,^18^. Interestingly, studies of lymphatic valve formation have shown that the ECM proteins fibronectin and laminin are deposited during the initial stages of valve development^11^,^19^, implicating ECM deposition in the earliest stages of the valve-forming process. The complex three-dimensional (3D) shape and constant motion of the heart, however, make imaging the morphogenetic events during cardiac valve development particularly challenging, although live imaging approaches are being continuously pioneered to observe endothelial cell behaviours in their mechanically active context^20^,^21^,^22^,^23^.

In the heart, the atrioventricular (AV) valve emanates from the endocardial wall and is composed of endocardial cells (EdCs) and ECM components^12^. While blood flow has a broad influence on the shape and growth of EdCs^6^, the oscillatory flow profile specific to the early AV canal (AVC) directs AV valve (AVV) formation by specifically increasing Krüppel-like factor 2a (*klf2a*) expression in the AVC^24^,^25^. As a transcription factor, *klf2a* expression likely allows EdCs to couple mechanotransduction to valve morphogenesis by activating a range of downstream target genes. The identity of such Klf2a target genes in valve-forming EdCs and the subsequent cellular behaviours induced, however, are unknown.

In this study, we investigated the cellular events taking place during valve formation and addressed their regulation by the flow-responsive transcription factor Klf2a. We show that valve formation proceeds via an initial stage of cell clustering followed by the appearance of cellular extensions towards the cardiac jelly. Subsequent global tissue remodelling events result in the appearance of ventricular and AVC-derived EdCs in the cardiac jelly overlying atrial-derived EdCs exposed to the lumen. Using transcriptomic analyses to highlight the transcriptional changes accompanying these temporally coordinated cell-movement events, we identified *fibronectin1b* as a key Klf2a- and flow-dependent factor necessary for the correct coordination of valvulogenesis. These data describe cell behaviour that is coordinated by the mechanical environment and mechanotransduction via Klf2a and ECM deposition.

## Results

### Endocardial cell contributions to the atrioventricular valve

**Figure 1:**
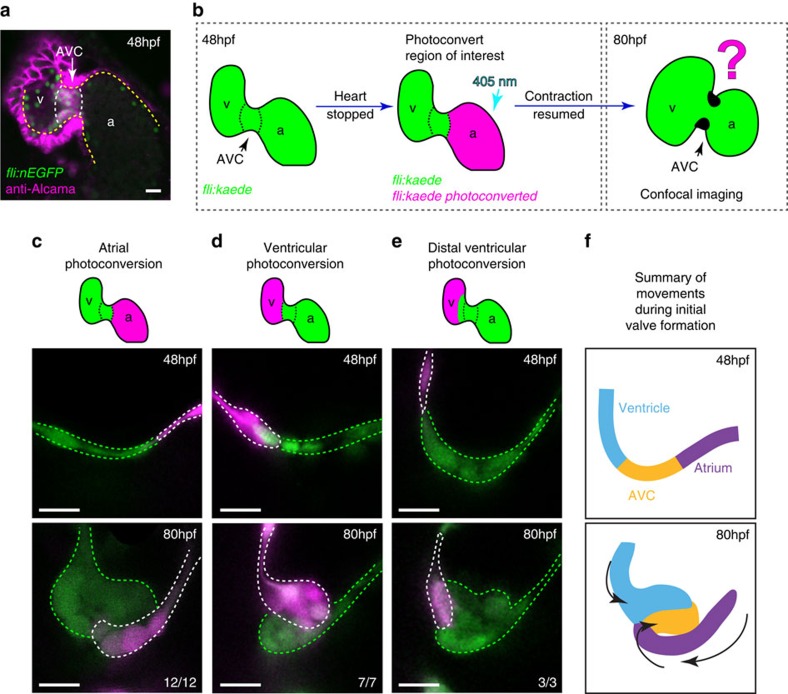
The cellular contribution of heart chambers to emerging valve leaflets. (**a**) anti-Alcama immunofluorescence analysis shows Alcama-positive EdCs in the AVC of *fli:nEGFP* hearts (white dotted line), used to define the cardiac chambers at 48 hpf. Yellow dashed line delineates the endocardium from the myocardium, which also stains positively for Alcama. v=ventricle, a=atrium, AVC=atrioventricular canal. (**b**) The experimental set-up for the photoconversion studies. Heart contraction was stopped in *fli:kaede* embryos at 48 hpf, the region of interest exposed to 405 nm light to convert kaede from a green to red (shown here to be the atrium, in magenta) fluorescent form and heart contraction was then resumed until 80 hpf. Stopped hearts were imaged at 80 hpf by confocal microscopy. (**c**) EdCs present in the atrium and Alcama-negative at 48 hpf lined the lumen of the AVC at 80 hpf (*n*=12/12, 3 experiments), while those in the ventricle contribute cells to the cardiac jelly (**d**; *n*=7/7, 3 experiments). Ventricular cells outside the ventricular inner curvature (distal ventricle) do not enter the cardiac jelly at 80 hpf (**e**; *n*=3/3, 2 experiments). In all cases only the superior valve leaflet is shown. (**f**) Schematic representation of the cellular contributions of each chamber to the emerging valve leaflet at 80 hpf. Atrial cells (purple) line the lumen of the AVC, while EdCs originating in the ventricular inner curvature (blue) and AVC (yellow) contribute cells to the cardiac jelly. Black arrows highlight the coordinated movements of the groups of cells. Scale bars, 10 μm.

AVV morphogenesis begins ∼48 hours post fertilization (hpf). By 5 days post fertilization (dpf) a set of functional valve leaflets, extend into the AVC, occluding the passage of reversing blood flow^26^,^27^,^28^. To uncover the origins of the EdCs contributing to the AVV, we performed photoconversion experiments using the *Tg(fli1a:Gal4FF*^*ubs*^*, UAS:kaede)* transgenic line, in which the photoconvertible protein kaede is expressed in the endothelial cells, including the endocardium. The exposure of kaede to 405 nm light results in an irreversible fluorescence conversion from fluorescent green to fluorescent red, enabling the development of cells labelled with the red form to be followed with respect to their green neighbours during AVV formation. As EdCs of the AVC can be identified by their positivity for Alcama^26^, we used our knowledge of this staining pattern (Fig. 1a) to specifically photoconvert green kaede to its red form in the atrium and ventricle at 48 hpf. We then focused on the subsequent development of the superior AVC as it undergoes valve morphogenesis earlier than the inferior AVC^26^. Heart contraction was temporarily blocked using 2,3-butanedione-2-monoxime (BDM) to enable the photoconversion to be performed. Following photoconversion, heart contraction was resumed and embryos were allowed to develop under standard conditions until imaging at 80 hpf, enabling us to assess the contribution of EdCs from each region to the forming superior AVV leaflet (Fig. 1b). Atrial cells photoconverted at 48 hpf were seen lining the AVC lumen at 80 hpf (Fig. 1c), and were never present inside the cardiac jelly following the formation of multiple cell layers (*n*=12/12). In contrast, photoconversion of cells in the ventricular region of the superior AVC at 48 hpf resulted in photoconverted cells in the cardiac jelly at 80 hpf (*n*=7/7 Fig. 1d). When ventricular photoconversion was performed away from the ventricular inner curvature, no photoconverted cells were observed inside the cardiac jelly (*n*=3/3; Fig. 1e). Thus, in addition to the cells of the AVC, EdCs from the atrium and ventricular inner curvature make significant and distinct contributions to the forming valve leaflets at 80 hpf (Fig. 1f). Furthermore, these distinct contributions are maintained at 120 and 168 hpf, suggesting mixing of cells from atrial and ventricular origins does not occur in the AVV at later stages (Supplementary Fig. 1). These findings implicate the ventricle and atrium as important sources of valve progenitors and suggest that stereotyped and coordinated cellular behaviours guide valve morphogenesis.

### Cell density and protrusive activity in early valvulogenesis

**Figure 2:**
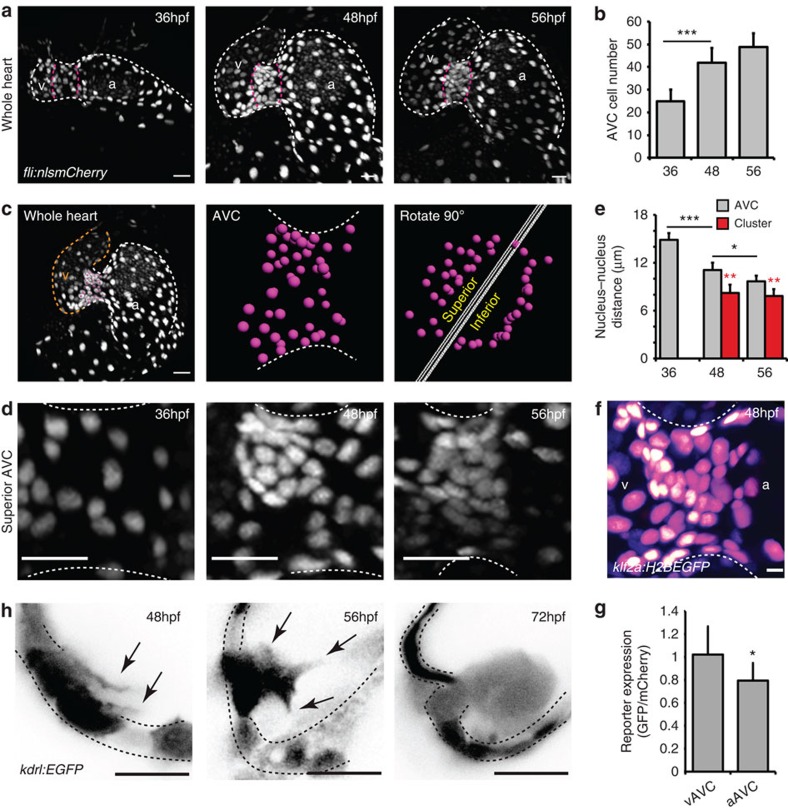
EdCs move into the cardiac jelly from a region of increased cell density. (**a**) *fli:nlsmCherry* hearts imaged at early stages of heart development show the organization of EdCs between 36 and 56 hpf. White dotted lines outline the endocardium. Magenta dotted lines mark the AVC. (**b**) Total AVC cell number quantification at 36, 48 and 56 hpf. (**c**) Demonstration of how the superior AVC is defined and presented in **d**: The nuclei of AVC EdCs are defined (magenta spots) from images of the whole heart (orange/v= ventricle; white/a=atrium), rotated 90 °C using Imaris software and then divided into two parts, the superior AVC (to be the upper leaflet) and the inferior AVC (to be the lower leaflet). Orthogonal views of the superior AVC are then shown to display nuclei organization in **d**. (**d**) Regional increases in cell density can be seen on the ventricular side of the AVC from 48 hpf (*n*=5 at each developmental stage). (**e**) Quantification of nucleus-nucleus distances within the AVC shows enhanced proximity of nuclei within the cluster region. No red bar at 36 hpf signifies the absence of a cluster at this stage. (**f**) *klf2a* expression pattern in the whole AVC at 48 hpf in the *klf2a:H2BEGFP* transgenic line shows enrichment of GFP signal on the ventricular side. The GFP signal is shown as FireLUT to aid visualization (white=highest intensity, black=no signal). (**g**) Relative reporter expression level in the ventricular (vAVC) and atrial (aAVC) regions of the AVC (*n*=10). (**h**) Imaging of *kdrl:EGFP* hearts demonstrates the presence of protrusions (black arrows) extending from the region of clustered cells towards the cardiac jelly at 48 hpf. Cells emanating into the cardiac jelly can be seen from 56 hpf. Protrusions are also still visible (black arrows). Groups of cells are present in the cardiac jelly by 72 hpf. Inverted images of the *kdrl:EGFP* signal are shown to aid visualization of the protrusive structures. Black dotted lines mark the EdCs layer. Error bars in all graphs represent the s.d. Student's *t*-test ***P<0.005, **P<0.01, *P<0.05. Scale bars, 10 μm; except in **f**: 2 μm.

To elucidate how cells of the atrium and ventricle reorganize to contribute to the developing valve leaflets, we characterized the organization of EdCs in the AVC in the moments preceding the appearance of multiple cell layers, beginning at 36 hpf. Using *Tg(fli:nlsmCherry)* embryos, in which the EdC nuclei are labelled, we observed nuclei to be relatively evenly spaced around the AVC at 36 hpf, before undergoing regional increases in cell density at 48 hpf (Fig. 2a–d and Supplementary Movie 1). Quantification of total cell numbers in the AVC showed a doubling of EdCs in the AVC between 36 and 48 hpf, (Fig. 2b). Photoconversion experiments suggest that cells move towards the AVC from the atrium between 36 and 48 hpf, while ventricular cells maintain their position at the ventricular inner curvature/exit of the AVC (Supplementary Fig. 2). In the absence of multi-layering, at this stage, this results in an increased density of cells within the AVC, particularly on the ventricular side. Incubation of embryos in BrdU between these stages demonstrated that ∼60% of the atrial cells and ∼40% of the cells from both the ventricle and the AVC proliferate during this time (Supplementary Fig. 2E,F), suggesting cell proliferation throughout the heart could be an important contributing factor to the increased cell density observed in the AVC at 48 hpf. Visualization of the superior leaflet alone enabled the changes in cell density between 36 and 48 hpf to be seen more clearly (Fig. 2c,d). A region of increased cell density was particularly apparent on the ventricular side of the superior AVC at 48 hpf, containing 11±3 cells (*n*=5). The numbers of cells in this clustered region stayed the same at 56 hpf (11±2 cells; *n*=5; Fig. 2d and Supplementary Movie 2). Quantification of distances between neighbouring nuclei at these stages confirmed cells in the clustered region were indeed more closely packed together than those around the rest of the AVC (Fig. 2e and see Supplementary Movie 3) suggesting regional increases in cell density accompany the increases in cell numbers observed.

We recently demonstrated the sensitivity of EdCs in detecting flow forces and inducing the expression of the flow-responsive transcription factor *klf2a* (ref. 24). In light of this, we investigated the distribution of *klf2a* expressing cells, more specifically, within the AVC. Using a *klf2a* reporter line (*Tg(klf2a:H2BEGFP)*^24^, we observed higher levels of GFP expression in those nuclei closest to the ventricle, compared with those on the atrial side of the AVC (Fig. 2f,g), corresponding to the region of cell clustering. Interestingly, protrusions were observed emanating from EdCs in this region towards the cardiac jelly at 48 hpf (Fig. 2h and Supplementary Movie 4). By 56 hpf, cells could be observed extending further into the cardiac jelly and by 72 hpf multiple layers of cells were present. To confirm the relevance of these observations in the beating heart, we performed fast confocal imaging of *Tg(kdrl:EGFP)* embryos and observed the same arrangement of cells at 72 hpf (*n*=3; Supplementary Movie 5). Indeed when the heart is contracting, the connection of the EdCs within the cardiac jelly to the region of the heart wall from where they originate is clear (Supplementary Movie 5). These observations enable us to describe, for the first time, a cluster of EdCs close to the ventricular inner curvature at 48 hpf, from which cellular protrusions and movement of cells into the cardiac jelly originates.

### Cardiac jelly cells present distinct characteristics

**Figure 3:**
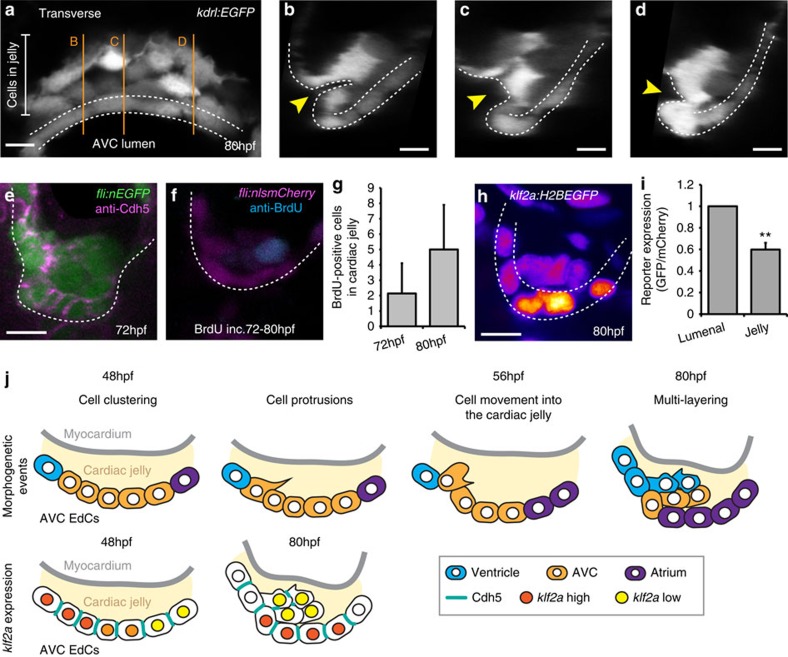
EdCs display distinct characteristics inside the cardiac jelly. (**a**) Transverse section of a *kdrl:EGFP* AVC at 80 hpf shows distinctive cell shapes within the AVC. Cells in the cardiac jelly have a mesenchymal morphology compared with those lining the lumen (enclosed in white dotted lines). Orange lines mark the positions of images i–iii. (**b**–**d**) white dotted line outlines the luminal EdCs and the yellow arrowheads highlight the deformation of the ventricular wall towards the cardiac jelly. The protrusive morphology of the cells within the cardiac jelly can also be observed. (**e**) Immunofluorescence analysis shows downregulation of Cdh5 between EdCs in the cardiac jelly (*n*=9). (**f**) anti-BrdU immunofluorescence following BrdU incubation between 72–80 hpf shows a BrdU-positive cell in the cardiac jelly. Shown is a single *z* slice. (**g**) Quantification of total numbers of BrdU-positive cells in the cardiac jelly at 72 hpf (BrdU incubation=56–72 hpf; *n*=7) and 80 hpf (BrdU incubation=72–80 hpf; *n*=5) (**h**) Imaging of the *klf2a:H2BEGFP* reporter line shows the *klf2a* expression pattern in the AVC at 80 hpf (GFP signal shown as FireLUT). (**i**) Normalized relative reporter expression levels in the EdCs exposed to the AVC lumen (lumenal) and those inside the cardiac jelly (jelly) (all cells in the superior AVC analysed from three embryos). Error bar represents the standard deviation. **P<0.01. (**j**) Model of the early stages of AVV formation. In the first series, cells are colour-coded to describe the morphogenetic events occurring during valve formation. Cells originating in the atrium (purple), AVC (yellow) and ventricle (blue) at 48 hpf and their relative positions at 80 hpf are shown. In the second series, Cdh5 and *klf2a* levels within the AVC are represented for the time points assessed. The myocardium is shown in grey and the cardiac jelly in pale yellow. Descriptions of the key stages at the relevant developmental time points are noted. Student's *t*-test ***P<0.005, **P<0.01, *P<0.05. Scale bars, 10 μm.

Analysis of the *Tg(kdrl:EGFP)* line at 80 hpf showed cells in the cardiac jelly to have a distinct mesenchymal-like morphology in comparison with those exposed to the lumen (Fig. 3a). Closer inspection of AVC morphology highlighted a deformation in the endocardial wall towards the cardiac jelly, which was wider towards the centre of the AVC (Fig. 3a–d). This morphology suggests a bending of the endocardial wall in response to the localized movement of EdCs into the cardiac jelly. In keeping with their mesenchymal-like morphology, junctional Cdh5 (VE-Cadherin) was lost between the neighbouring cells in the cardiac jelly at 72 hpf (Fig. 3e). Interestingly, BrdU-incorporation assays revealed the presence of BrdU-positive cells in the cardiac jelly, but not in the AVC wall between 56–72 hpf and 72–80 hpf (Fig. 3f and g). This suggests EdCs proliferate in the cardiac jelly, but do not enter it as a result of asymmetric cell division. Finally, analysis of *klf2a* reporter activity at 80 hpf, after multi-layering, showed EdCs exposed to the lumen expressed higher levels of *klf2a* activity than those in the cardiac jelly (Fig. 3h,i). This suggests *klf2a* is not continuously expressed in the EdCs that undergo multi-layering, as they originate in the region of the AVC where *klf2a* expression is high and then enter the cardiac jelly where *klf2a* expression is low. Furthermore, cells originally expressing low levels of *klf2a* in the atrium^24^ appear to initiate *klf2a* expression upon entering the AVC. These observations define two subsets of cells within the AVC following multi-layering; one *klf2a*^low^, Cdh5^low^, proliferative population within the cardiac jelly overlying a second *klf2a*^high^, Cdh5^high^ population exposed to the blood flow. On the basis of these observations we propose a model to describe the early cellular events involved in AVV formation (Fig. 3j). EdCs cluster in a region of the AVC close to the ventricular inner curvature at 48 hpf, corresponding to the region of increased *klf2a* expression. EdCs in this clustered region extend protrusions and emanate into the cardiac jelly, initiating the coordinated morphogenetic movements that result in multiple layers of EdCs within the cardiac jelly by 80 hpf. Once inside the cardiac jelly, EdCs display a mesenchymal-like phenotype with reduced levels of Cdh5 and *klf2a*, and increased proliferation.

### Transcriptional changes in early valvulogenesis

**Figure 4:**
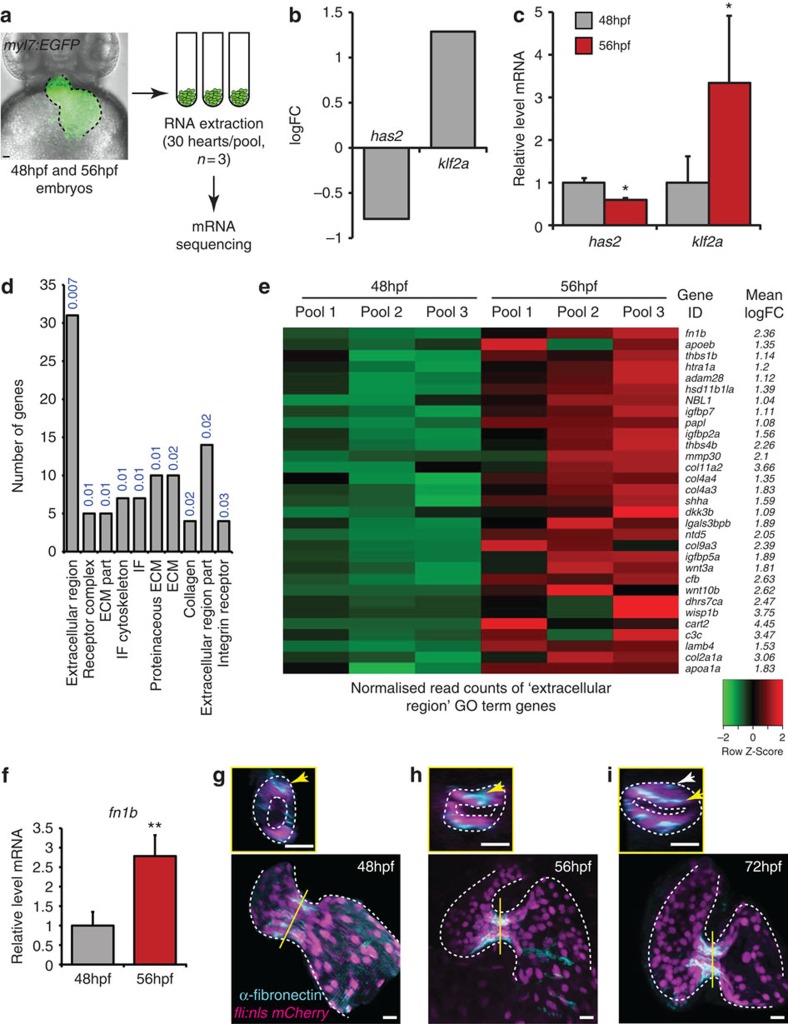
Transcriptome analysis identifies increased ECM protein gene expression during initial stages of valve formation. (**a**) Experimental set-up for mRNA sequencing. RNA was extracted from hearts of *myl7:EGFP* embryos dissected at 48 and 56 hpf, in pools of 30 (*n*=3). (**b**) Downregulation of *has2* and upregulation of *klf2a* transcripts, confirmed by qPCR (**c**), validated our approach. (**d**) Gene Ontology (GO) analysis based on cellular compartment terms of upregulated genes highlighted a significant enrichment of ECM-related terms. *P* values describing the significance of each term enrichment are shown in blue. (**e**) Specific analysis of the most significantly enriched group (the ‘extracellular region' term), highlighting, among others, elevated fibronectin1b expression between 48 and 56 hpf. (**f**) qPCR analysis of heart RNA confirms an increase in fn1b transcript levels between 48 and 56 hpf. (**g**–**i**) Immunofluorescence analysis shows fibronectin synthesis is localized to the AVC during early stages of valve formation (*n*=8 (48 hpf), *n*=11 (56 hpf) and *n*=7 (72 hpf)). Yellow lines mark the position of the transverse sections shown in small panels. Yellow arrows highlight enriched fibronectin-positive staining in the superior AVC. White arrow in small panel (**i**) points to fibronectin deposition on multiple cell layers. Student's *t*-test ***P<0.005, **P<0.01, *P<0.05. Scale bars, 10 μm.

To elucidate how these early events are regulated, we sought to determine the gene expression profile activated at these early stages of valve formation. To do so, we extracted RNA from hearts dissected from 48 and 56 hpf *myl7:EGFP* embryos and performed transcriptome analysis using an Illumina sequencing platform (Fig. 4a). Between 31 and 65 million reads were generated for each RNA sample, of which, on an average, 67% could be mapped onto the Zv9 assembly of the zebrafish genome. We identified 1,628 genes that were significantly, differentially expressed in the heart between 48 and 56 hpf (FDR<0.05). Importantly, we saw a downregulation of *has2* and an upregulation of *klf2a*, confirming the reliability of our approach (Fig. 4b). Real-time quantitative PCR (qPCR) analysis of heart RNA confirmed the respective up and downregulation of these genes with time (Fig. 4c). Considering only those genes with a logFC>1, we found 1,076 genes to be upregulated during these early stages of valve development. To assess the biological significance of these genes, we then performed Gene Ontology analysis using DAVID software^29^. By clustering genes based on cellular compartment annotations we saw a strong enrichment for ECM protein terms (Fig. 4d). Closer analysis of the 31 genes found in the ‘extracellular region' term demonstrated that, among others, *fibronectin1b* was significantly upregulated in the heart between 48 and 56 hpf (Fig. 4e). Given the importance of fibronectin deposition in lymph valve formation^19^, we questioned whether it could also play a role in AVV formation. qPCR analysis of heart-derived RNA showed fibronectin1b is indeed expressed in the heart during this period of its development and confirmed the increase of transcript levels between 48 and 56 hpf (Fig. 4f). We next addressed the temporal and spatial expression of fibronectin within the heart at the protein level through immunofluorescence analysis. Interestingly, fibronectin protein is expressed specifically in the AVC at 48 hpf (Fig. 4g) with further enrichment by 56 hpf, in keeping with the mRNA sequencing data (Fig. 4h). More specifically, fibronectin is seen on the basal side of cells at both time points and is particularly enriched in the region of cell clustering (Fig. 4g,h). Fibronectin can also be observed on and between the multiple layers of cells that are present by 72 hpf (Fig. 4i), as has previously been shown in 105 hpf hearts^26^. This spatial and temporal expression pattern in the heart suggests a potentially relevant role of *fibronectin1b*/fibronectin in cardiac valve development.

### Blood flow and *klf2a* alter fibronectin synthesis in the AVC

**Figure 5:**
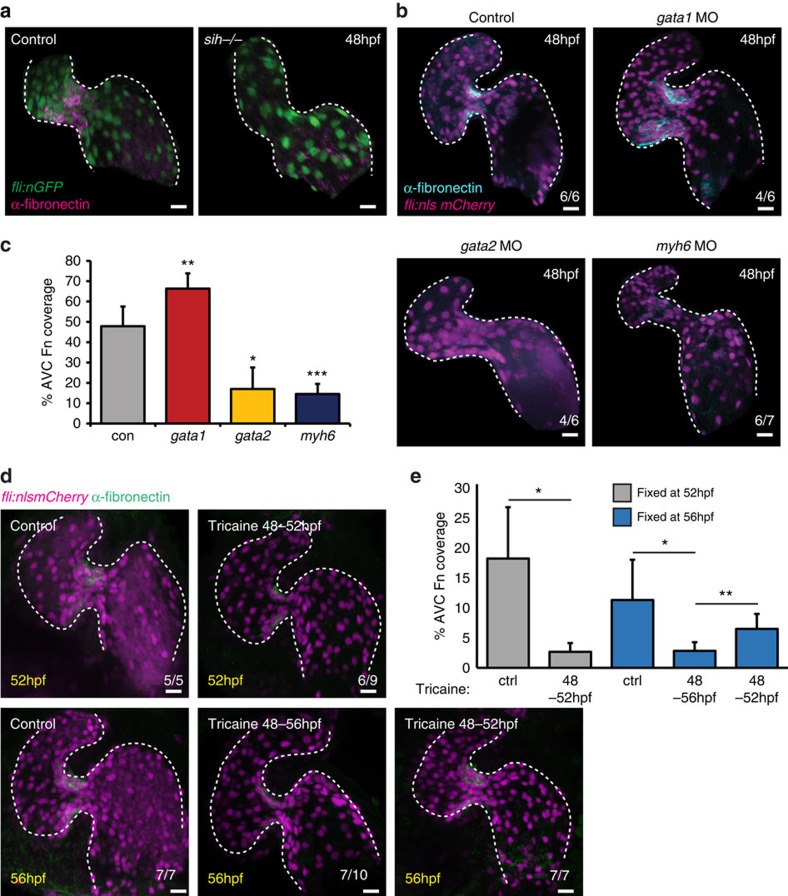
AVC-specific fibronectin synthesis is dependent on blood flow forces. (**a**) Fibronectin staining (magenta) is lost in the absence of heart contraction (*fli:nEGFP, sih*^*−/−*^ embryos) (*n*=6/6). (**b**) *fli:nlsmCherry* embryos injected with *gata1*, *gata2* or *myh6* morpholinos to alter blood flow forces were fixed at 48 hpf and anti-fibronectin immunofluorescence analysis was performed. Alterations in flow forces impacted fibronectin synthesis (cyan) in the AVC of *gata1*, *gata2* or *myh6* morphants. (**c**) Quantification of extent of fibronectin (Fn)-positive staining confirms an increase in *gata1* morphants and a reduction in both *gata2* and *myh6* morphants. (**d***) fli:nlsmCherry* (magenta) embryos were incubated with 0.1% tricaine between 48–52 and 48–56 hpf to stop heart contraction. When necessary, heart contraction was restored at 52 hpf by washing out tricaine and embryos were left to develop under normal conditions until 56 hpf. Yellow font shows embryonic age at fixation. Anti-fibronectin immunofluorescence (green) shows the flow-responsive nature of fibronectin in the AVC. (**e**) Quantification of extent of fibronectin-positive staining confirms the restoration of fibronectin synthesis following restoration of heart contraction. Control (ctrl) samples were not incubated in 0.1% tricaine. Student's *t*-test ***P<0.005, **P<0.01, *P<0.05. Scale bars, 10 μm.

As blood flow is an important regulator of EdC behaviour and cardiac valve formation, we next wanted to assess whether changes in flow properties impacted fibronectin synthesis in the AVC. To do so, we first analysed the fibronectin staining pattern in silent heart (*sih*^*−/−*^) mutant embryos, which completely lack heart contraction and blood flow^30^, and saw that fibronectin was no longer detectable in the AVC at 48 hpf (Fig. 5a). As *sih*^*−/−*^ mutants fail to form an AVV, we performed photoconversion experiments following injection of a morpholino specific for *troponin T2a* (*tnnt2a*), which is necessary for heart contraction and reliably mimics the *sih*^*−/−*^ mutants^30^, to determine whether the cell-movement events described above were impacted in the absence of heart contraction. Indeed, in *tnnt2a*MO hearts at 80 hpf, the photoconverted cells were found on the inner curvature of the ventricle and had failed to enter the cardiac jelly, as observed in age-matched controls (Supplementary Fig. 3A,B). To address the role of flow forces more specifically, we then altered blood viscosity and shear stress by lowering haematocrit content by injecting *gata1* and *gata2* morpholinos, as previously described^25^. In *gata1* morphants, where the fraction of reversing flow in the AVC at 48 hpf is increased and *klf2a* expression is high^24^, strong fibronectin staining was observed in the AVC (Fig. 5b), while it was much reduced or absent in *gata2* morphants where the fraction of reversing flow in the AVC, and *klf2a* expression, is reduced (Fig. 5b). When atrial contraction was affected in *myh6* morphants (atrial specific myosin heavy chain, previously *amhc*), which also results in reduced *klf2a* expression^31^ (Supplementary Fig. 3E,F), fibronectin deposition was also impaired (*n*=6/7; Fig. 5b). Quantification of the proportion of the AVC positive for fibronectin confirmed these observations (Fig. 5c). Furthermore, fibronectin synthesis was significantly reduced, compared with controls, when 0.1% tricaine was used to stop heart contraction between 48 and 52 hpf and between 48 and 56 hpf (Fig. 5d). Interestingly, when the 0.1% tricaine was removed and heart contraction resumed at 52 hpf, fibronectin staining in the AVC at 56 hpf was restored (Fig. 5d,e). Taken together these observations suggest that the synthesis of fibronectin in the AVC is flow-dependent. To ascertain how conditions of altered flow may impact cellular organization during valve formation, we repeated our photoconversion experiments in the flow morphants described above. We observed cells in the cardiac jelly of *gata1* MO embryos, but this was greatly reduced in *gata2* MO and absent in *myh6* MO embryos, when compared with controls (Supplementary Fig. 3C,D). Furthermore, in *gata1* MOs, cells in the cardiac jelly appeared to be more disorganized than in controls (Supplementary Fig. 3C,D) suggesting AVC-specific fibronectin synthesis may be necessary for the correct organization of multiple cell layers before leaflet emanation.

### AVC-specific fibronectin synthesis is necessary for valve formation

**Figure 6:**
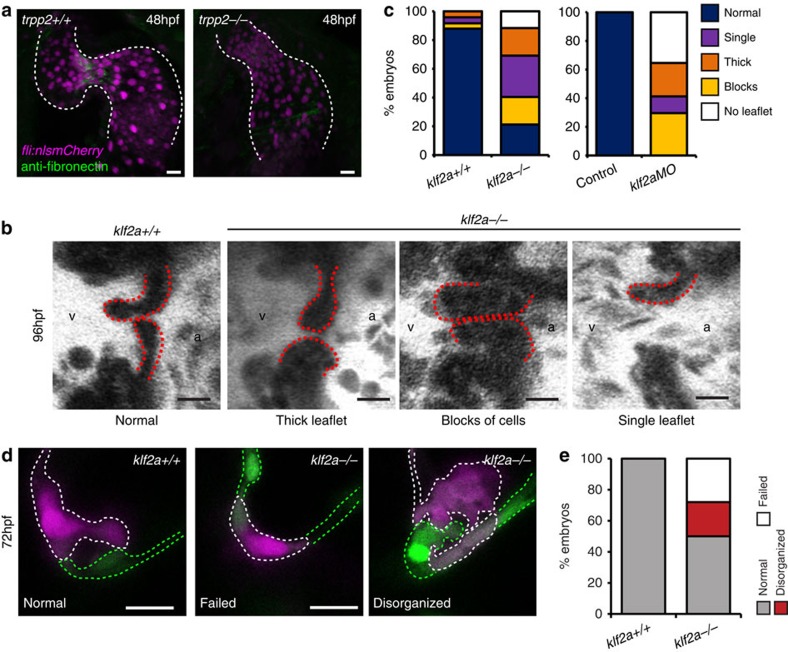
Characterization of *klf2a*^*−/−*^ mutants. (**a**) anti-fibronectin staining (green) is reduced in the AVC of *trpp2*^*−/−*^ mutants (12/12 *trpp2*^*+/+*^, 9/19 *trpp2*^*−/−*^). (**b**,**c**) A range of valvular defects were observed in *klf2a*^*−/−*^ mutant embryos at 96 hpf (*n*=25 *klf2a*^*+/+*^, *n*=48 *klf2a*^*−/−*^), similar to those observed in *klf2a*MO-injected embryos (**e**; *n*=20 control, *n*=17 *klf2a*MO). (**d**) Photoconversion of AVC EdCs at 48 hpf shows the organization of cells within the cardiac jelly to be affected by loss of *klf2a* (**d**,**e**; *n*=21 *klf2*^*+/+*^, *n*=32 *klf2a*^*−/−*^) at 72 hpf. Scale bars, 10 μm.

Considering the apparent flow-dependent nature of fibronectin synthesis in the AVC and its increased levels on the ventricular side of the superior AVC, where *klf2a* is most highly expressed, we reasoned that *fibronectin1b* could be a downstream target of Klf2a. As *klf2a* is elevated in response to the specific flow regime found within the AVC^24^, such a target would enable *klf2a* to both respond to the mechanical environment of the AVC and impact the local environment of EdCs in a manner necessary for valve formation. Indeed, fibronectin staining was reduced in *trpp2*^*−/−*^ mutants (Fig. 6a), which present defects in valvulogenesis and *klf2a* induction despite a normal flow regime in the AVC^24^. To address the effect of loss of *klf2a* and validate previous observations performed using morpholino-based approaches, we generated a mutant of *klf2a* using a TALEN approach targeting a sequence in the first exon of the *klf2a* gene (Supplementary Fig. 4A). Observations of valve morphology at 96 hpf, when valve leaflets can be seen extending into the lumen of the AVC in controls, demonstrated a range of valvular defects in *klf2a* mutants (Fig. 6b,c; *n*=25 wild-type, *n*=46 *klf2a*^*−/−*^) despite there being no change in overall cell numbers (Supplementary Fig. 4B) or flow properties (Supplementary Fig. 4E) at 48 hpf. Approximately 10% of *klf2a*^*−/−*^ embryos were missing any kind of valve structure. Identical analyses performed in *klf2a* morpholino-injected embryos showed a similar, but more severe, phenotype in the knock down (Fig. 6c) and all subsequent studies were performed with the *klf2a* mutant. To investigate origins of the valve defects observed in the *klf2a*^*−/−*^ mutants, we examined these embryos during the early stages of valve formation described above. Analysis of cell organization showed *klf2a*^*−/−*^ embryos had fewer cells clustering together in the superior AVC at 48 hpf than controls (Supplementary Fig. 4C). Quantification of cells within the cardiac jelly suggested multi-layering was impaired in ∼40% of *klf2a*^*−/−*^ hearts while the remaining *klf2a*^*−/−*^ hearts presented elevated numbers of EdCs in the cardiac jelly at 72 hpf when compared with controls (Fig. 6d and Supplementary Fig. 4D). In those mutants where multi-layering occurred, however, the cells in the cardiac jelly appeared disorganized, with intracellular spaces between neighbouring EdCs, compared with the compact nature of the cells in this area in controls (Fig. 6d,e and Supplementary Movie 6). These data suggest that, in the absence of *klf2a* function, the cellular processes underlying the initiation of valve formation are perturbed and support observations made with *klf2a* morpholinos that *klf2a* expression is necessary for efficient valvulogenesis^25^.

**Figure 7:**
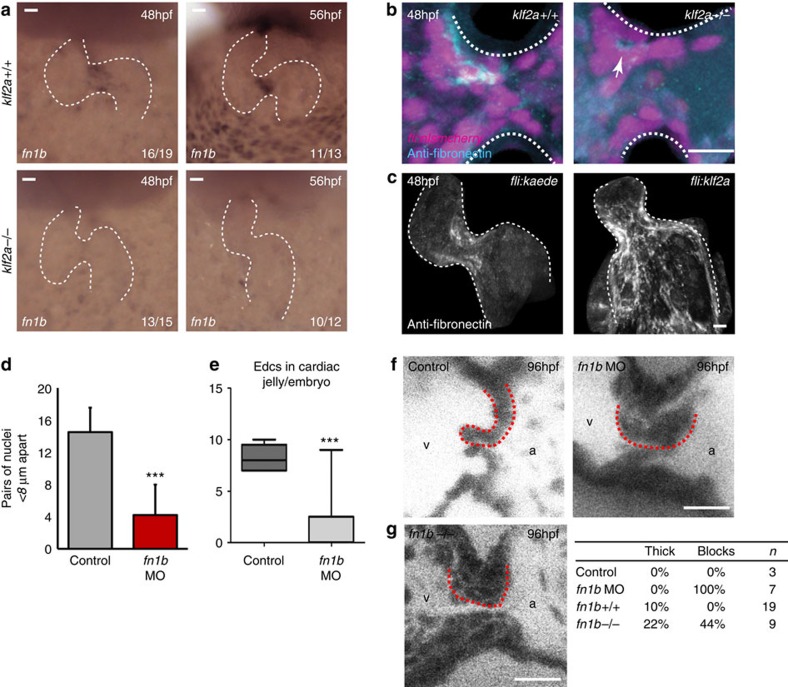
Klf2a expression regulates fibronectin synthesis in the AVC to drive valve formation. (**a**) *in situ* hybridization analysis shows the *fn1b* mRNA expression in the AVC of *klf2a*^*+/+*^ embryos, at 48 and 56 hpf, is lost in the majority of *klf2a*^*−/−*^ embryos. (**b**) Immunofluorescence analysis shows reduced fibronectin-positive staining (cyan) in the AVC of *klf2a*^*−/−*^ mutants (*n*=12 *klf2*^*+/+*^, *n*=18 *klf2a*^*−/−*^). A *z*-projection of the whole AVC is shown. (**c**) Forced expression of Klf2a in EdCs using the *Tg(fli:gal4FF*^*ubs*^*; UAS:klf2a)* line results in fibronectin-positive staining in the atrium and ventricle (*n*=12/16) compared with the AVC-specific staining pattern in controls (*n*=12/14) in at least three independent experiments. A *z*-projection of the whole heart is shown. (**d**) Analysis of *fn1b* morphants showed defective cell clustering in the superior AVC at 48 hpf (control *n*=6, *fn1b*MO *n*=5) and (**e**) reduced numbers of nuclei in the cardiac jelly at 72 hpf (control *n*=5, *fn1b*MO *n*=9). (**f**) Live imaging of valves at 96 hpf showed leaflets fail to form in *fn1b* morphants (controls *n*=3, *fn1b*MO *n*=7). Single frames from the live imaging are shown and red dotted lines outline the superior valve leaflet in each case. The ventricle (v) and atrium (a) are labelled for orientation. (**g**) Single frames from the live imaging of *fn1b*^*−/−*^ mutant hearts, quantification of the percentage of thick/blocked valves observed in *fn1b* MO, *fn1b*^*−/−*^ mutant hearts and their respective controls. Student's *t*-test ***P<0.005, **P<0.01, *P<0.05. Scale bars, 10 μm.

*In situ* hybridization and immunofluorescence analysis demonstrated that fibronectin is downregulated at both the mRNA and protein level in the majority of klf2a mutants (Fig. 7a,b). Furthermore, when we forced the overexpression of *klf2a* in all endothelial cells, we saw a spread of fibronectin synthesis outside the AVC, into the atrium and ventricle, in comparison with the AVC-specific localization observed in controls (Fig. 7c). This suggests that Klf2a, the expression of which is normally restricted to the AVC, is capable of driving the expression of fibronectin in the heart. Indeed, forced expression of *klf2a* was sufficient to rescue fibronectin synthesis in *gata2* and *myh6* morphant embryos (Supplementary Fig. 5G). To quantify the efficacy of valve progenitors in undergoing multi-layering in the absence of fibronectin, a vital process in the formation of the heart valve, we used a *fn1b*-specific morpholino to deplete fibronectin in the AVC (Supplementary Fig. 5A). Care was taken to select embryos presenting no morphological defects following *fn1b*MO injection (Supplementary Fig. 5B), ensuring that *klf2a* reporter activity, AVC cell number and the flow velocity profile at 48 hpf (Supplementary Fig. 5D–F), as well as levels of p53 mRNA (Supplementary Fig. 5C), were not significantly changed in the morphants studied. Cell clustering and multi-layering were dramatically impaired in the absence of *fn1b*, with few or no cells in the cardiac jelly in the majority of cases (Fig. 7d,e) indicating the importance of fibronectin deposition in the early stages of valve development. At 96 hpf, large groups of EdCs and reversing blood flow were visible in the AVC of *fn1b*MO embryos in contrast to the efficient valve leaflets present in controls (Fig. 7f). To confirm these observations, we analysed the valve shape of *fn1b*^*sa553−/−*^ embryos at 96 hpf. Similar to *fn1b* knock down, we found that all the fn1b mutants had abnormal valves (9/9). The majority of these embryos displayed large blocks of cells occluding the AVC or thick leaflets as sometimes also described in the *klf2a* mutants (Fig. 7g). These data suggest *klf2a* and *fn1b* expression are necessary for the movement of EdCs into the cardiac jelly and their correct organization within. Thus mechanically-induced *klf2a* expression coordinates the morphogenetic events necessary for valve formation via the regulation of *fibronectin1b* expression and localized fibronectin synthesis within the AVC.

## Discussion

Using *in vivo* imaging technologies and cellular scale 3D analysis, we have identified three successive steps highlighting the cellular processes associated with heart valve morphogenesis: (1) the first signs of valve formation correspond to a regional increase in cell density in the superior AVC and localized fibrillogenesis; (2) the first sign of cellular invasion towards the cardiac jelly is highlighted by cell protrusions specifically in this area of cell clustering; and (3) a highly stereotyped multi-layering process within the cardiac jelly leading to the formation of a functional leaflet. These observations enable us to confirm the previously reported role of mechanical forces in valve morphogenesis^10^,^25^ and suggest a refined model in which the origins of the valve progenitors, the behaviour of particular groups of cells and the impact of the mechanotransduction cascade are identified.

Clustering of EdCs in a region of the AVC close to the ventricular inner curvature and localized fibronectin synthesis in the same area at 48 hpf is followed by the appearance of cells protruding into the cardiac jelly by 56 hpf. In the absence of *klf2a*, an important component of the mechanotransduction pathway downstream of blood flow^24^, cell clustering and fibrillogenesis are both impaired. Ultimately, the coordinated morphogenetic movements subsequently observed in control embryos are perturbed in *klf2a* mutants highlighting the importance of an intact mechanotransduction pathway in orchestrating the cellular events involved in valve formation. Interestingly, we observed a striking change in cell properties following migration into the cardiac jelly, with cells demonstrating a mesenchymal morphology, downregulating junctional Cdh5 and some of them proliferating. While we cannot rule out a contribution of cell proliferation in other parts of the heart driving cells towards the AVC, within the AVC itself we only observed cell proliferation within the cardiac jelly and not within the cell layer that is exposed to the blood flow. We demonstrated that flow forces primarily influence cell behaviour and ultimately valve shape, but not cell number, via the asymmetric activation of *klf2a* expression in the ventricular region of the AVC. Interestingly, *klf2a*^*−/−*^ mutants presented an array of valvular phenotypes similar to those observed under a range of altered flow regimes, implicating Klf2a as an integrator of the flow response, which, when absent, impacts valvulogenesis from the earliest stages. It will be interesting to see if fibronectin also relays Klf2a function in other contexts, such as haematopoietic stem cells (HSC) formation^32^, development of the branchial arches^33^ and endocardial chamber ballooning^6^.

During valve formation the cells lining a unicellular tube are required to undergo extensive rearrangements in order to form a protrusive 3D structure capable of occluding undesired reversing flow. In the lymphatic system, the initial phases of valve formation have been elegantly described proceeding via the clustering of lymphatic valve progenitors on one side of the collecting vessel, adoption of a cuboidal morphology and the generation of a ring-like constriction within the vessel wall^11^. Valve-forming cells then protrude into the lumen of the lymphatic vessels, which, accompanied by ECM deposition, results in the formation of a valve leaflet^11^,^19^. In an initially similar manner, we see EdCs clustering on the ventricular side of the superior AVC, but rather than protruding towards the lumen, clustered EdCs then send basal protrusions into the cardiac jelly that they subsequently invade. These cells then proliferate and form a group of cells nestled close to the ventricular inner curvature, before a clear leaflet structure is formed. This significant difference in acquisition of cells into the leaflet in the lymphatic and cardiac systems is likely to be, at least in part, attributable to the different mechanical environments experienced by their progenitors.

During cardiac valve formation in the mouse, EdCs within the AVC undergo an endothelial-to-mesenchymal transition (EMT) and migrate into the cardiac jelly^34^, where they proliferate and valve leaflets subsequently elongate. This is in striking contrast to lymphatic valve formation, which proceeds in the absence of an EMT. Interestingly, by embryonic day 10.5 (E10.5) the loss of *klf2* results in AVC cell disorganization and hypocellular cushions, attributed to a defect in the EMT process^35^. We also observe AVC cell disorganization and, in some instances, hypocellular cushions in the absence of *klf2a*. Considering this together with the downregulation of Cdh5 by EdCs inside the cardiac jelly, cell morphology changes and increased proliferation, we hypothesize that the cellular protrusions observed from the clustered cells from 48 hpf and the subsequent movement of cells into the cardiac is reminiscent of the valve-forming process described in the mouse^35^. Importantly, the presence of an EMT during zebrafish valvulogenesis, although extensively discussed^18^,^26^,^28^,^36^, remains to be confirmed and will be an important focus of future studies.

Valve morphogenesis occurs in one of the most hostile mechanical environments in the body: EdCs experience both high flow forces and strong mechanical deformation due to the contraction of the heart and its associated blood flow^1^. Indeed, at embryonic stages, viscosity dominates and the main mechanical forces generated at the heart wall are the tissue strain generated by pressure variations occurring during heart contraction and wall shear stress generated by the flowing blood^1^. These physical features are crucial to understanding how EdCs behave during valve development. Cell clustering close to the ventricular inner curvature in the AVC suggests localized differences in cell tension, which in turn may be responsible for the localized fibrillogenesis observed before multi-layering.

The specific enrichment of *klf2a* expression in the AVC at 48 hpf in response to flow and its regulation of *fibronectin1b* expression provides a mechanism to modulate valve morphogenesis in response to its mechanical environment^25^. For now, we can only speculate on the role of fibronectin in the process of valve formation. One possibility is that fibronectin is necessary for EdCs to acquire a valvular cell fate as it is now becoming clear that forces, ECM and stem cells fate are tightly interdependent^37^. The discovery that the mechanical environment and stretch-sensitive channels are essential determinants of lineage choices in embryonic stem cells further supports this hypothesis^38^,^39^,^40^. Fibronectin could also be involved in promoting the generation of the filopodia observed at the onset of AVC-specific fibronectin accumulation and before the multi-layering process. Indeed, fibronectin-rich nanoenvironments have been demonstrated to be sufficient for orienting cell migration and proliferation^41^. In addition, fibronectin may alter the mechanical properties of the EdCs environment as well as several cellular properties, including the mechanosensitivity of the cells themselves. Fibronectin has been implicated in the endothelial mechanotranduction pathway mediated by *trpv4* (ref. 42), a gene that is also involved in AVV formation^24^. Alternatively, fibrillogenesis could participate in cell-shape changes associated with morphogenesis^43^, a feature that has also been attributed to *klf2a* expression^6^.

In conclusion, we propose a model for AVV formation in which EdCs arising from the atrium and ventricle make specific contributions to the emerging valve leaflets through coordinated cell-movement events. Enriched mechanosensitive *klf2a* expression on the ventricular side of the AVC subsequently enriches the expression of its downstream target gene *fibronectin1b* to the same cells, establishing an asymmetry within the AVC. Ventricular EdCs extend protrusions towards the fibronectin-rich area and move into the cardiac jelly in a coordinated manner while accompanying morphogenetic changes result in atrial EdCs lining the lumen of the AVC. Subsequent Cdh5-downregulation, increased proliferation and morphological changes within the AVC liken aspects of AVV formation in the zebrafish to that of the mouse and higher vertebrates.

Finally, this study elucidates the impact of mechanical forces on the localized behaviours of valve progenitors during valve morphogenesis. As the origins of most valvulopathies are still unknown^12^, our findings highlight the importance of investigating the potential embryonic and mechanical origins of valve defects. The recent demonstration that mitral valve disease can have embryonic origins supports this view^44^,^45^,^46^. Furthermore our work highlights the role of the coordination between morphogenesis and mechanical forces via ECM synthesis during cardiovascular morphogenesis. These findings may also prove meaningful in other biological contexts where mechanical forces and ECM are involved, such as during stem cell niche formation in developing HSCs^47^,^48^,^49^, skin stem cells^50^ and adult intestinal stem cells^51^.

## Methods

### Zebrafish husbandry, embryo treatments and morpholinos

Animal experiments were approved by the Animal Experimentation Committee of the Institutional Review Board of the IGBMC. Zebrafish lines used in this study were *Tg(fli1a:gal4FF*^*ubs*^*; UAS:kaede)*^52^, *Tg(fli1a:nEGFP)*^*y7*^ (ref. 53)*, Tg(kdrl:EGFP)*^54^*, Tg(fli:nls-mcherry)*^24^*, Tg(myl7:EGFP)*^55^*, Tg(fli:gal4FF*^*ubs*^*; UAS:klf2a)* (ref. 6), *Tg(klf2a:H2BEGFP)*^24^ and wild-type AB. Zebrafish with a mutant allele of *klf2a (Tg(klf2a*^*ig4*^*))* were generated and used in this study. Zebrafish with a mutant allele of *fn1b (Tg(fn1b*^*Sa553*^*)* were obtained from the Zebrafish International Resource Center (ZIRC). *Tg(fn1b*^*Sa553*^) mutants contain a C>T point mutation in exon 1 of the *fn1b* gene leading to a premature stop codon in the predicted translation product. Genotyping was performed by sequencing the PCR product generated with the following primers: forward 5′- AGGGTGAGAGAACCTCATAAAGC -3′, reverse 5′- CTCACTTAAACCGCGAACTGTCC -3′, and sequenced with 5′- TCAGTAAAGAGACTCCTGCTGC -3′. Genotyping was performed on genomic DNA after live imaging. Morpholinos were injected into the yolk at the one-cell stage. All animals were incubated at 28.5 °C for 5 h before treatment with 1-phenyl-2-thiourea (PTU) (Sigma Aldrich) to prevent pigment formation. Morpholinos specific for *tnnt2a* (ref. 30) (5′- CATGTTTGCTCTGATCTGACACGCA -3′) and *fn1b* (5′- AAGTAATAATGTCACCTTGCTCCTC -3′) were obtained from GeneTools. Morpholinos for *klf2a*, *gata1, gata2* and *myh6* were described previously^25^,^56^. Anti-sense MO concentrations ranged from 0.06 to 0.3 mM.

### Generation of *klf2a* ^*−/−*^ mutants

We injected a TALEN pair designed to target exon 1 of the *klf2a* gene into single cell wild-type (AB) embryos. We identified the alleles generated and confirmed that potential targeting events could be transmitted through the germline by out-crossing the F0 fish with AB animals and sequencing genomic DNA from pools of 6 F1 embryos. We focused on an INDEL mutation (deletion of 5′- CAGAAGGAA -3′ followed by insertion of 5′- GATGCTGGGAGAG -3′) leading to a premature stop codon in the *klf2a* transcript and raised these F1 animals to adulthood. Studies were performed on F2 fish, and later generations, following out-crossing to transgenic lines of interest. *Klf2a*^*−/−*^ fish were viable and also kept as homozygous adults. A PCR-based genotyping strategy was established using the following primers to identify the wild-type and mutant alleles (Wild-type: forward 5′- TCGGCGCAGAAGGAAA -3′, reverse 5′- TGTTGAGGTTGTCCATGTTA -3′; mutant: forward 5′- AAGGTCTTCCACCACTCATA -3′, reverse 5′- CCAGCATTTCTCTCCCAGC -3′). Genotyping was performed on genomic DNA from whole embryos after live imaging, or from dissected tails before immunofluorescence analysis, as necessary.

### mRNA sequencing of dissected heart samples

Hearts were dissected from *Tg(myl7:EGFP)* embryos at the desired stage and pooled (30 hearts/sample). RNA was extracted using a Nucleospin RNA XS kit (Macherey-Nagel) according to the manufacturer's instructions. After isolation of total cellular RNA, a library of template molecules suitable for high throughput DNA sequencing was created following the Illumina ‘mRNA sequencing sample preparation guide' (part #1004898 Rev.D) with some modifications. Briefly, mRNA was purified from 20 ng total RNA using oligo-dT magnetic beads and fragmented using divalent cations at 94 °C for 5 min. The cleaved mRNA fragments were reverse transcribed to cDNA using random primers, then the second strand of the cDNA was synthesized using DNA Polymerase I and RNase H. The next steps of RNA-Seq Library preparation were performed in a fully automated system using SPRIworks Fragment Library System I kit (ref A84801, Beckman Coulter, Inc) with the SPRI-TE instrument (Beckman Coulter, Inc). Briefly, in this system, double-stranded cDNA fragments were blunted, phosphorylated and ligated to indexed adapter dimers, and fragments in the range of ∼200–400 bp were size selected. The automated steps were followed by PCR amplification (30 s at 98 °C; (10 s at 98 °C, 30 s at 60 °C and 30 s at 72 °C) × 13 cycles; 5 min at 72 °C), then surplus PCR primers were removed by purification using AMPure XP beads (Agencourt Biosciences Corporation). DNA libraries were checked for quality and quantified using a 2100 Bioanalyzer (Agilent). The libraries were loaded in the flow cell at 6 pM concentration and clusters generated and sequenced in the Illumina Genome Analyzer IIX as single-end 54 base reads. The data discussed in this publication have been deposited in NCBI's Gene Expression Omnibus^57^ and are accessible through GEO Series accession number GSE79585 ( https://www.ncbi.nlm.nih.gov/geo/query/acc.cgi?acc= GSE79585).

### Bioinformatics and gene ontology analysis

Read quality was assessed with FastQC (S. Andrews, http://www.bioinformatics.babraham.ac.uk/projects/fastqc/). Then reads were mapped onto the Zv9 assembly of the zebrafish genome using Tophat v1.4.1 (ref. 58) and the bowtie v0.12.7 aligner. Only uniquely aligned reads were retained for further analyses. Gene expression was quantified using HTSeq v0.5.3p5 (ref. 59) and gene annotations from Ensembl release 69. Read counts were normalized across libraries with the method proposed by Anders *et al*.^60^. To identify significantly differentially expressed genes, we performed a test for differential expression within the experiment (that is, adjusting for baseline differences between the experiments) using the method proposed by Robinson and Smyth^61^ and implemented in the Bioconductor edgeR v3.0.8 package^62^. Adjustment for multiple testing was performed with the Benjamini and Hochberg^63^ method. Functional analyses of genes with an adjusted *P* value smaller than 0.05 were performed using DAVID software^64^. Graphics were obtained with the R program (R Core Team, URL http://www.R-project.org/).

### qPCR

Products were amplified in a real-time PCR reaction with Light Cycler 480 Real-Time PCR System (Roche) using a UPL Probes Master mix (Roche) according to the manufacturer's instructions. Sequence of primer pairs were as follows: *has2* forward 5′- AGCATCCCTGTTCAACTAACG -3′, reverse 5′- GCTGACCGCTTTATCACATCT -3′*; klf2a* forward 5′- CCGTCTATTTCCACATTTTCG -3′, reverse 5′- TCCAGTTCATCCTTCCACCT -3′; *fn1b* forward 5′- TGGAAATGTGATGCCATTGA -3′, reverse 5′- GGCCAATCTGGTAGAACACC -3′; *p53* forward 5′- GAGGTCGGCAAAATCAATTC -3′, reverse 5′- TGGGGGCTGAATAATCAAAT -3′.

### *In vivo* imaging

Zebrafish embryos were staged, anaesthetised with 0.02% tricaine solution and 50 mM BDM, to stop the heart when necessary, and mounted in 0.7% low melting-point agarose (Sigma Aldrich). Confocal imaging was performed on a Leica SP8 confocal microscope. Fast confocal and four-dimensional imaging (to image valve leaflets at 96 hpf and the AVC at 72 hpf) was performed using the resonant scanner mode of the same microscope. Images were acquired with a low-magnification water immersion objective (Leica HCX IRAPO L, 25X, N.A. 0.95). For four-dimensional imaging, time series were acquired at a random time in the cardiac cycle at 35fps for 3 s. The optical plane was moved 2 μm between the *z*-sections until the whole AVC was acquired. Time series of two-dimensional sections were temporally synchronized using Matlab^27^. Blood flow imaging for flow velocity analysis was performed on a Leica DMIRBE inverted microscope using a Photron SA3 high speed CMOS camera (Photron, San Diego, CA) and water immersion objective (Leica × 20, NA 0.7). Image sequences were acquired at a frame rate of 2,000 frames per second.

### Photoconversion

Photoconversion was performed using the FRAP module on a SP8 confocal microscope and a Leica HCX IRAPO L, × 25, NA0.95 water immersion objective. *Tg(fli1a:Gal4FF; UAS:Kaede)* embryos were mounted in 0.7% low melting-point agarose supplemented with 50 mM BDM to inhibit heart contraction for the duration of the procedure. A region of interest corresponding to the ventricle, atrium or superior AVC was selected and exposed to 405 nm light (25% laser power). One pre-bleach frame was acquired, followed by 3–5 bleach pulses (3–5 ms each) without acquisition to achieve conversion of the kaede protein to its red form. A *z*-stack of the photoconverted heart was then acquired in the standard confocal mode to record the starting point of each experiment. Embryos were then carefully dissected from the agarose, placed in fish water for 5–10 min until heart contraction resumed and then put at 28.5 °C to develop individually under standard conditions until a time point of interest. The movement of cells within each heart was analysed using Imaris software (Bitplane).

### Valve imaging

Embryos were incubated with 4 μM BODIPY-ceramide (Molecular Probes) overnight and then processed as in refs 24, 25 to visualize the valve shape.

### Flow analysis

Red blood cells were manually tracked through the AVC and their velocity calculated from image sequences acquired at 2,000 frames per second as described previously^24^.

### Immunofluorescence

Embryos were fixed at the desired stage in 2% paraformaldehyde overnight at 4 °C. BrdU-incorporation studies were performed by incubating embryos in 5 mg ml^−1^ BrdU for the desired length of time (Dietrich *et al*.^6^) before fixation. After washing, embryos were permeabilized in 1 × PBS-0.1% Tween-20 containing 0.5% Triton-X 100 for 30 min at room temperature. The pericardial cavity was then carefully pierced with the tip of a forcep to facilitate antibody penetration before blocking in permeabilization buffer supplemented with 5% BSA (anti-fibronectin), 1% BSA and 10% NGS (anti-VECadherin and anti-Alcama) or 1% BSA, 2% NGS and 1% DMSO (anti-BrdU) for 2 h at room temperature. Primary antibodies were added in the relevant blocking solution and incubated between 16 and 48 h at 4 °C. Secondary antibodies were added in blocking solution after thorough washing and incubated overnight at 4 °C. Embryos were thoroughly washed and mounted for imaging on a Leica SP8 confocal. Antibodies were as follows: rabbit anti-fibronectin (F3648, Sigma) 1:100, rabbit anti-VECadherin^65^ 1:1,000 (kind gift of the Affolter lab), mouse anti-BrdU (11170376001, Roche Diagnostics) 1:100, mouse anti-Alcama (zn-8, DSHB) 1:500 and goat anti-rabbit and goat anti-mouse Alexa-488 and -594 secondary antibodies (A11034 and A11032, respectively, Life Technologies) were used at 1:500. To directly test the effects of flow on fibronectin synthesis, embryos were incubated in 0.1% tricaine (Ethyl 3-aminobenzoate methanesulfonic acid; Sigma) at pH7 for up to 12 h. They were then rinsed briefly in egg water before being fixed and processed as described above.

### Cell proliferation assay

Dechorionated embryos were incubated in fish water containing 5 mg ml^−1^ BrdU between 36 and 48 hpf, 56 and 72 hpf, and 72 and 80 hpf. Incorporation was stopped by washing in fresh fish water and fixation in 4% PFA. Embryos were permeabilized with Proteinase K and DNA denatured with 2 N HCl (method modified from Dietrich *et al*.^6^). BrdU immunolabelling was then performed as described above.

### Image analysis

Cell number quantifications and *klf2a*:*EGFP* signal intensity measurements were made using the Spots tool on Imaris (Bitplane). A single spot was placed at the centre of each nucleus in the AVC, or cardiac jelly, as appropriate. For intensity analysis, the *klf2a:H2BEGFP* reporter line was crossed with the *fli:nlsmCherry* line and the mCherry fluorescence signal was used for normalization^24^. The maximum intensity of each channel was quantified and a ratio generated. These ratios were then averaged across the AVC of individual embryos. Nucleus to nucleus distance analysis was performed using Imaris software and the Measurement Points tool. Nuclei within the AVC were connected to all of their nearest neighbours and the average distances for defined regions were calculated. The extent of cell clustering, in *klf2a* mutants and *fn1b* morphants, was quantified by defining clustered cells as those cells closer than 8 μm to their neighbour (according to our analysis of nucleus-to-nucleus distances in controls; Fig. 2e). The extent of fibronectin staining in the AVC was calculated using the Imaris Surfaces tool to define a volume of fibronectin staining and a volume of the whole AVC. The % of fibronectin coverage was then calculated ([Volume fibronectin staining/Volume AVC]*100). Hearts were segmented using the surfaces tool and the segmented heart presented, for clarity.

### *In situ* hybridization

*In situ* hybridization was performed as in ref. 66. Anti-sense probes for *fn1b* were generated from a plasmid containing *fn1b* cDNA (obtained from SourceBioscience) amplified using the following primers forward: 5′- ATGACCCGTGAGTCAGTAA -3′ and reverse (containing T3 sequence): 5′- ATTAACCCTCACTAAAGGGACTTGGTGCCCTGAGTTCTGAT -3′ and subsequently transcribed using the T3 polymerase.

## Additional information

**Accession codes:** The gene expression data have been deposited in the NCBI Gene Expression Omnibus (GEO) database under accession code GSE79585.

**How to cite this article:** Steed, E. *et al*. *klf2a* couples mechanotransduction and zebrafish valve morphogenesis through Fibronectin synthesis. *Nat. Commun.* 7:11646 doi: 10.1038/ncomms11646 (2016).

## Supplementary Material

Supplementary Information Supplementary Figures 1-5

Supplementary Movie 1 Endocardial cell organization at 36hpf and 48hp.*Tg(fli:nlsmCherry)* hearts were imaged at 36hpf and 48hpf. Magenta spots mark nuclei of the AVC. Yellow spots mark nuclei in the AVC cluster.

Supplementary Movie 2 Endocardial cell organization at 56hpf and 72hpf.*Tg(fli:nlsmCherry)* hearts were imaged at 56hpf and 72hpf. Magenta spots mark nuclei of the AVC. Yellow spots mark nuclei in the AVC cluster. Cyan spots mark nuclei in the cardiac jelly.

Supplementary Movie 3 Method for analyzing nuclei spacing within the AVC. Imaris software (Bitplane) was used to place a spot at the center of each nucleus within the AVC of *Tg(fli:nlsmCherry)* embryos acquired by confocal microscopy. White lines connect nuclei within the AVC using the "Measurement points" tool and the distances between nuclei were obtained.

Supplementary Movie 4 Cell protrusions towards the cardiac jelly at 48hpf. Three-dimensional volume of a *Tg(kdrl:EGFP)* 48hpf heart shows a cell extending a protrusion towards the cardiac jelly at from the ventricular side of the AVC. Segmentation of the protrusive cell is shown in yellow.

Supplementary Movie 5 Live imaging of kdrl:EGFP at 72hpf. Synchronisation of a 72hpf beating *Tg(kdrl:EGFP)* heart acquired at 35fps. The "Oblique slicer" tool on Imaris software shows the level of the AVC at which cells can be seen extending in to the cardiac jelly from the AVC/ventricular inner curvature region (yellow arrow and label). A single slice from the synchronized movie is then played to demonstrate the motion of these EdCs within the cardiac jelly during heart contraction. Movie is played back at 10fps.

Supplementary Movie 6 Cell organization in the cardiac jelly in *klf2a+/+* and *klf2a-/-* hearts. Z-stacks of *klf2a+/+* (top) and *klf2a-/-* (bottom) *fli:kaede* AVCs shows the organization of cells at 72hpf. The AVCs were photoconverted at 48hpf. Photoconverted cells are shown in magenta, non-photoconverted cells are shown in green.

## References

[b1] Boselli F., Freund J. B. & Vermot J. Blood flow mechanics in cardiovascular development. Cell. Mol. Life Sci. 72, 2545–2559 (2015). 25801176 10.1007/s00018-015-1885-3 PMC4457920

[b2] Freund J. B., Goetz J. G., Hill K. L. & Vermot J. Fluid flows and forces in development: functions, features and biophysical principles. Development 139, 1229–1245 (2012). 22395739 10.1242/dev.073593 PMC3294433

[b3] Heisenberg C. P. & Bellaiche Y. Forces in tissue morphogenesis and patterning. Cell 153, 948–962 (2013). 23706734 10.1016/j.cell.2013.05.008

[b4] Guillot C. & Lecuit T. Mechanics of epithelial tissue homeostasis and morphogenesis. Science 340, 1185–1189 (2013). 23744939 10.1126/science.1235249

[b5] Mammoto T. & Ingber D. E. Mechanical control of tissue and organ development. Development 137, 1407–1420 (2010). 20388652 10.1242/dev.024166 PMC2853843

[b6] Dietrich A. C., Lombardo V. A., Veerkamp J., Priller F. & Abdelilah-Seyfried S. Blood flow and Bmp signaling control endocardial chamber morphogenesis. Dev. Cell 30, 367–377 (2014). 25158852 10.1016/j.devcel.2014.06.020

[b7] Liu J. . A dual role for ErbB2 signaling in cardiac trabeculation. Development 137, 3867–3875 (2010). 20978078 10.1242/dev.053736 PMC3049280

[b8] Peshkovsky C., Totong R. & Yelon D. Dependence of cardiac trabeculation on neuregulin signaling and blood flow in zebrafish. Dev. Dyn. 240, 446–456 (2011). 21246662 10.1002/dvdy.22526

[b9] Peralta M. . Heartbeat-driven pericardiac fluid forces contribute to epicardium morphogenesis. Curr. Biol. 23, 1726–1735 (2013). 23954432 10.1016/j.cub.2013.07.005

[b10] Hove J. R. . Intracardiac fluid forces are an essential epigenetic factor for embryonic cardiogenesis. Nature 421, 172–177 (2003). 12520305 10.1038/nature01282

[b11] Sabine A. . Mechanotransduction, PROX1, and FOXC2 cooperate to control connexin37 and calcineurin during lymphatic-valve formation. Dev. Cell. 22, 430–445 (2012). 22306086 10.1016/j.devcel.2011.12.020

[b12] MacGrogan D. How to make a heart valve: from embryonic development to bioengineering of living valve substitutes. Cold Spring Harb. Perspect. Med. 4, a013912 (2014). 25368013 10.1101/cshperspect.a013912 PMC4208706

[b13] Wallingford J. B. Planar cell polarity and the developmental control of cell behavior in vertebrate embryos. Annu. Rev. Cell. Dev. Biol. 28, 627–653 (2012). 22905955 10.1146/annurev-cellbio-092910-154208

[b14] Back M., Gasser T. C., Michel J.-B. & Caligiuri G. Biomechanical factors in the biology of aortic wall and aortic valve diseases. Cardiovasc. Res. 99, 232–241 (2013). 23459103 10.1093/cvr/cvt040 PMC3695745

[b15] Sauls K. . Developmental basis for filamin-A-associated myxomatous mitral valve disease. Cardiovasc. Res. 96, 109–119 (2012). 22843703 10.1093/cvr/cvs238 PMC3444235

[b16] Richards J. M., Farrar E. J., Kornreich B. G., Moïse N. S. & Butcher J. T. The mechanobiology of mitral valve function, degeneration, and repair. J. Vet. Cardiol. 14, 47–58 (2012). 22366572 10.1016/j.jvc.2012.01.002 PMC3586284

[b17] Odelin G. . Loss of Krox20 results in aortic valve regurgitation and impaired transcriptional activation of fibrillar collagen genes. Cardiovasc. Res. 104, 443–455 (2014). 25344368 10.1093/cvr/cvu233

[b18] Lagendijk A. K., Szabo A., Merks R. M. & Bakkers J. Hyaluronan: a critical regulator of endothelial-to-mesenchymal transition during cardiac valve formation. Trends Cardiovasc. Med. 23, 135–142 (2013). 23295082 10.1016/j.tcm.2012.10.002

[b19] Bazigou E. . Integrin-alpha9 is required for fibronectin matrix assembly during lymphatic valve morphogenesis. Dev. Cell 17, 175–186 (2009). 19686679 10.1016/j.devcel.2009.06.017 PMC2747264

[b20] Boselli F. & Vermot J. Live imaging and modeling for shear stress quantification in the embryonic zebrafish heart. Methods 94, 129–134 (2015). 26390811 10.1016/j.ymeth.2015.09.017

[b21] Huisken J. & Stainier D. Y. Selective plane illumination microscopy techniques in developmental biology. Development 136, 1963–1975 (2009). 19465594 10.1242/dev.022426 PMC2685720

[b22] Lenard A. . Endothelial cell self-fusion during vascular pruning. PLoS Biol. 13, e1002126 (2015). 25884426 10.1371/journal.pbio.1002126 PMC4401649

[b23] Mahou P., Vermot J., Beaurepaire E. & Supatto W. Multicolor two-photon light-sheet microscopy. Nat. Methods 11, 600–601 (2014). 24874570 10.1038/nmeth.2963

[b24] Heckel E. . Oscillatory flow modulates mechanosensitive klf2a expression through trpv4 and trpp2 during heart valve development. Curr. Biol. 25, 1354–1361 (2015). 25959969 10.1016/j.cub.2015.03.038

[b25] Vermot J. . Reversing blood flows act through klf2a to ensure normal valvulogenesis in the developing heart. PLoS Biol. 7, e1000246 (2009). 19924233 10.1371/journal.pbio.1000246 PMC2773122

[b26] Beis D. . Genetic and cellular analyses of zebrafish atrioventricular cushion and valve development. Development 132, 4193–4204 (2005). 16107477 10.1242/dev.01970

[b27] Liebling M. . Rapid three-dimensional imaging and analysis of the beating embryonic heart reveals functional changes during development. Dev. Dyn. 235, 2940–2948 (2006). 16921497 10.1002/dvdy.20926

[b28] Scherz P. J., Huisken J., Sahai-Hernandez P. & Stainier D. Y. High-speed imaging of developing heart valves reveals interplay of morphogenesis and function. Development 135, 1179–1187 (2008). 18272595 10.1242/dev.010694

[b29] Huang H. T. . A network of epigenetic regulators guides developmental haematopoiesis *in vivo*. Nat. Cell. Biol. 15, 1516–1525 (2013). 24240475 10.1038/ncb2870 PMC3959952

[b30] Sehnert A. J. . Cardiac troponin T is essential in sarcomere assembly and cardiac contractility. Nat. Genet. 31, 106–110 (2002). 11967535 10.1038/ng875

[b31] Kalogirou S. . Intracardiac flow dynamics regulate atrioventricular valve morphogenesis. Cardiovasc. Res. 104, 49–60 (2014). 25100766 10.1093/cvr/cvu186 PMC4271066

[b32] Wang L. . A blood flow-dependent klf2a-NO signaling cascade is required for stabilization of hematopoietic stem cell programming in zebrafish embryos. Blood 118, 4102–4110 (2011). 21849483 10.1182/blood-2011-05-353235

[b33] Nicoli S. . MicroRNA-mediated integration of haemodynamics and Vegf signalling during angiogenesis. Nature 464, 1196–1200 (2010). 20364122 10.1038/nature08889 PMC2914488

[b34] von Gise A. & Pu W. T. Endocardial and epicardial epithelial to mesenchymal transitions in heart development and disease. Circ. Res. 110, 1628–1645 (2012). 22679138 10.1161/CIRCRESAHA.111.259960 PMC3427736

[b35] Chiplunkar A. R. . Kruppel-like factor 2 is required for normal mouse cardiac development. PloS One 8, e54891 (2013). 23457456 10.1371/journal.pone.0054891 PMC3573061

[b36] Lagendijk A. K., Goumans M. J., Burkhard S. B. & Bakkers J. MicroRNA-23 restricts cardiac valve formation by inhibiting Has2 and extracellular hyaluronic acid production. Circ. Res. 109, 649–657 (2011). 21778427 10.1161/CIRCRESAHA.111.247635

[b37] Bellas E. & Chen C. S. Forms, forces, and stem cell fate. Curr. Opin. Cell Biol. 31, 92–97 (2014). 25269668 10.1016/j.ceb.2014.09.006 PMC4252586

[b38] Engler A. J., Sen S., Sweeney H. L. & Discher D. E. Matrix elasticity directs stem cell lineage specification. Cell 126, 677–689 (2006). 16923388 10.1016/j.cell.2006.06.044

[b39] McBeath R., Pirone D. M., Nelson C. M., Bhadriraju K. & Chen C. S. Cell shape, cytoskeletal tension, and RhoA regulate stem cell lineage commitment. Dev. Cell 6, 483–495 (2004). 15068789 10.1016/s1534-5807(04)00075-9

[b40] Trappmann B. . Extracellular-matrix tethering regulates stem-cell fate. Nat. Mater. 11, 642–649 (2012). 22635042 10.1038/nmat3339

[b41] Albuschies J. & Vogel V. The role of filopodia in the recognition of nanotopographies. Sci. Rep. 3, 1658 (2013). 23584574 10.1038/srep01658 PMC3625890

[b42] Thodeti C. K. . TRPV4 channels mediate cyclic strain-induced endothelial cell reorientation through integrin-to-integrin signaling. Circ. Res. 104, 1123–1130 (2009). 19359599 10.1161/CIRCRESAHA.108.192930 PMC2754067

[b43] Compagnon J. . The notochord breaks bilateral symmetry by controlling cell shapes in the zebrafish laterality organ. Dev. Cell 31, 774–783 (2014). 25535919 10.1016/j.devcel.2014.11.003

[b44] Dina C. . Genetic association analyses highlight biological pathways underlying mitral valve prolapse. Nat. Genet. 47, 1206–1211 (2015). 26301497 10.1038/ng.3383 PMC4773907

[b45] Durst R. . Mutations in DCHS1 cause mitral valve prolapse. Nature 525, 109–113 (2015). 26258302 10.1038/nature14670 PMC4720389

[b46] Levine R. A. . Mitral valve disease-morphology and mechanisms. Nat. Rev. Cardiol. 12, 689–710 (2015). 26483167 10.1038/nrcardio.2015.161 PMC4804623

[b47] Jing L. . Adenosine signaling promotes hematopoietic stem and progenitor cell emergence. J. Exp. Med. 212, 649–663 (2015). 25870200 10.1084/jem.20141528 PMC4419349

[b48] Kim P. G. . Flow-induced protein kinase A-CREB pathway acts via BMP signaling to promote HSC emergence. J. Exp. Med. 212, 633–648 (2015). 25870201 10.1084/jem.20141514 PMC4419355

[b49] Diaz M. F. . Biomechanical forces promote blood development through prostaglandin E2 and the cAMP-PKA signaling axis. J. Exp. Med. 212, 665–680 (2015). 25870199 10.1084/jem.20142235 PMC4419354

[b50] Hsu Y. C., Li L. & Fuchs E. Emerging interactions between skin stem cells and their niches. Nat. Med. 20, 847–856 (2014). 25100530 10.1038/nm.3643 PMC4358898

[b51] Shyer A. E., Huycke T. R., Lee C., Mahadevan L. & Tabin C. J. Bending gradients: how the intestinal stem cell gets its home. Cell 161, 569–580 (2015). 25865482 10.1016/j.cell.2015.03.041 PMC4409931

[b52] Herwig L. . Distinct cellular mechanisms of blood vessel fusion in the zebrafish embryo. Curr. Biol. 21, 1942–1948 (2011). 22079115 10.1016/j.cub.2011.10.016

[b53] Roman B. L. . Disruption of acvrl1 increases endothelial cell number in zebrafish cranial vessels. Development 129, 3009–3019 (2002). 12050147 10.1242/dev.129.12.3009

[b54] Jin S. W., Beis D., Mitchell T., Chen J. N. & Stainier D. Y. Cellular and molecular analyses of vascular tube and lumen formation in zebrafish. Development 132, 5199–5209 (2005). 16251212 10.1242/dev.02087

[b55] Huang C. J., Tu C. T., Hsiao C. D., Hsieh F. J. & Tsai H. J. Germ-line transmission of a myocardium-specific GFP transgene reveals critical regulatory elements in the cardiac myosin light chain 2 promoter of zebrafish. Dev. Dyn. 228, 30–40 (2003). 12950077 10.1002/dvdy.10356

[b56] Galloway J. L., Wingert R. A., Thisse C., Thisse B. & Zon L. I. Loss of gata1 but not gata2 converts erythropoiesis to myelopoiesis in zebrafish embryos. Dev. Cell 8, 109–116 (2005). 15621534 10.1016/j.devcel.2004.12.001

[b57] Edgar R., Domrachev M. & Lash A. E. Gene Expression Omnibus: NCBI gene expression and hybridization array data repository. Nucleic Acids Res. 30, 207–210 (2002). 11752295 10.1093/nar/30.1.207 PMC99122

[b58] Trapnell C., Pachter L. & Salzberg S. L. TopHat: discovering splice junctions with RNA-Seq. Bioinformatics. 25, 1105–1111 (2009). 19289445 10.1093/bioinformatics/btp120 PMC2672628

[b59] Anders S. HTSeq: Analysing high-throughput sequencing data with Python. Available at: http://www-huber.embl.de/users/anders/HTSeq (2014). 10.1093/bioinformatics/btac166 PMC9113351 35561197

[b60] Robinson M. D. & Oshlack A. A scaling normalization method for differential expression analysis of RNA-seq data. Genome Biol. 11, R25 (2010). 20196867 10.1186/gb-2010-11-3-r25 PMC2864565

[b61] McCarthy D. J., Chen Y. & Smyth G. K. Differential expression analysis of multifactor RNA-Seq experiments with respect to biological variation. Nucleic Acids Res. 40, 4288–4297 (2012). 22287627 10.1093/nar/gks042 PMC3378882

[b62] Robinson M. D., McCarthy D. J. & Smyth G. K. edgeR: a bioconductor package for differential expression analysis of digital gene expression data. Bioinformatics 26, 139–140 (2010). 19910308 10.1093/bioinformatics/btp616 PMC2796818

[b63] Benjamini Y. & Hochberg Y. Controlling the false discovery rate: a practical and powerful approach to multiple testing. J. R. Stat. Soc. 57, 289–300 (1995).

[b64] Huang, da W., Sherman B. T. & Lempicki R. A. Systematic and integrative analysis of large gene lists using DAVID bioinformatics resources. Nat. Protoc. 4, 44–57 (2009). 19131956 10.1038/nprot.2008.211

[b65] Blum Y. . Complex cell rearrangements during intersegmental vessel sprouting and vessel fusion in the zebrafish embryo. Dev. Biol. 316, 312–322 (2008). 18342303 10.1016/j.ydbio.2008.01.038

[b66] Thisse C. & Thisse B. High-resolution *in situ* hybridization to whole-mount zebrafish embryos. Nat. Protoc. 3, 59–69 (2008). 18193022 10.1038/nprot.2007.514

