# Supplementary material for: klf2a couples mechanotransduction and zebrafish valve morphogenesis through fibronectin synthesis
Source: Nat Commun. 2016 May 25;7:11646. doi: 10.1038/ncomms11646 (PMC4894956; doi:10.1038/ncomms11646)
Supplement: Supplementary Information — Supplementary Figures 1-5 [file ncomms11646-s1.pdf]

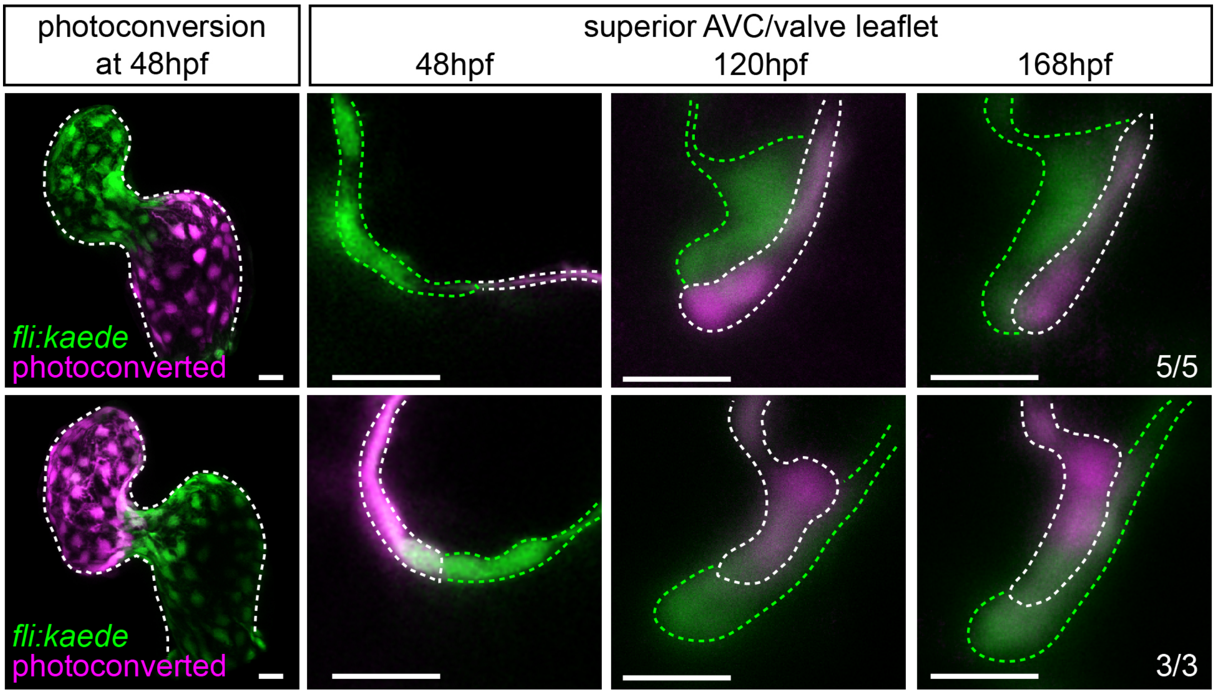

Supplementary Figure 1 – Contribution of atrial and ventricular cells to the AV valve leaflets at later stages. Endocardial cells of the atrium or ventricle were photoconverted at 48hpf as shown, and then allowed to develop normally. Hearts were briefly stopped for imaging at 120hpf and 168hpf. No mixing of atrial and ventricular cells was observed in the superior valve leaflet at these later stages. Atrial cells lined the atrial side of the valve leaflet at 120hpf and 168hpf, and ventricular cells lined the ventricular side. Images shown are from the same hearts at 48hpf, 120hpf and 168hpf. White-dotted lines mark the cells containing photoconverted kaede (magenta). Green-dotted lines mark the cells containing non-photoconverted kaede (green). Scale bars: 10 µm.

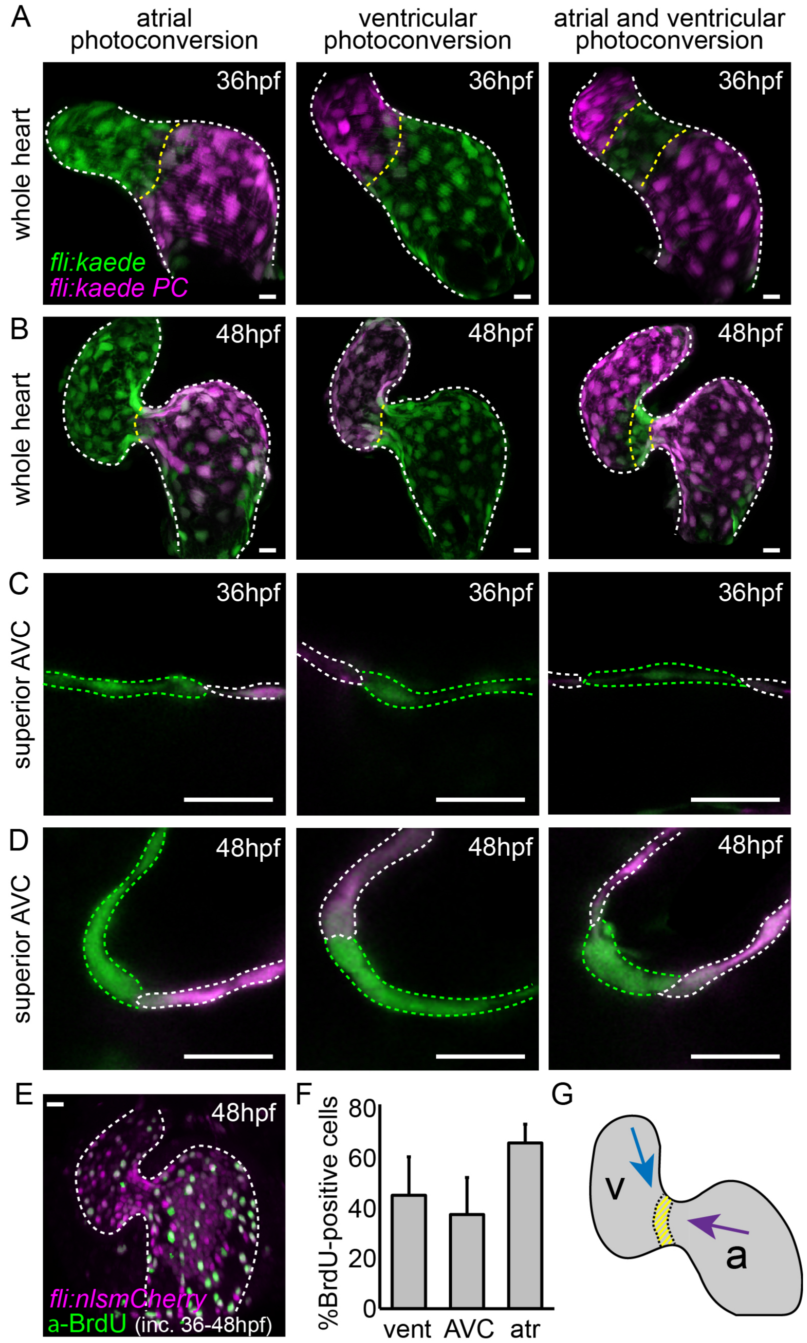

Supplementary Figure 2 – Cell movements and proliferation may contribute to increased AVC cell density at 48hpf. (A) Kaede-expressing cells in the atrium, ventricle, or both at 36hpf were photoconverted (photoconverted = magenta; non-photoconverted = green) and the hearts were allowed to develop normally until 48hpf. (B) Imaging stopped hearts at 48hpf showed photoconverted cells of the atrium to be in the AVC at 48hpf. Cells of the ventricle are present in the ventricular inner curvature at 48hpf. (E) Incubation of embryos with BrdU between 36hpf and 48hpf followed by anti-BrdU immunofluorescence analysis demonstrated the presence of BrdU-positive cells (green) in both chambers of the heart. (F) Quantification of BrdU-positive cells in the ventricle, AVC and atrium (n=8). (G) Representation of the movement of cells from the atrium (purple arrow) and the ventricle (blue arrow) between 36hpf and 48hpf. The yellow region represents cells from the AVC of the 36hpf heart, which cluster together on the ventricular side of the AVC at 48hpf. Error bar represents the standard deviation. Scale bars: 10 μm.

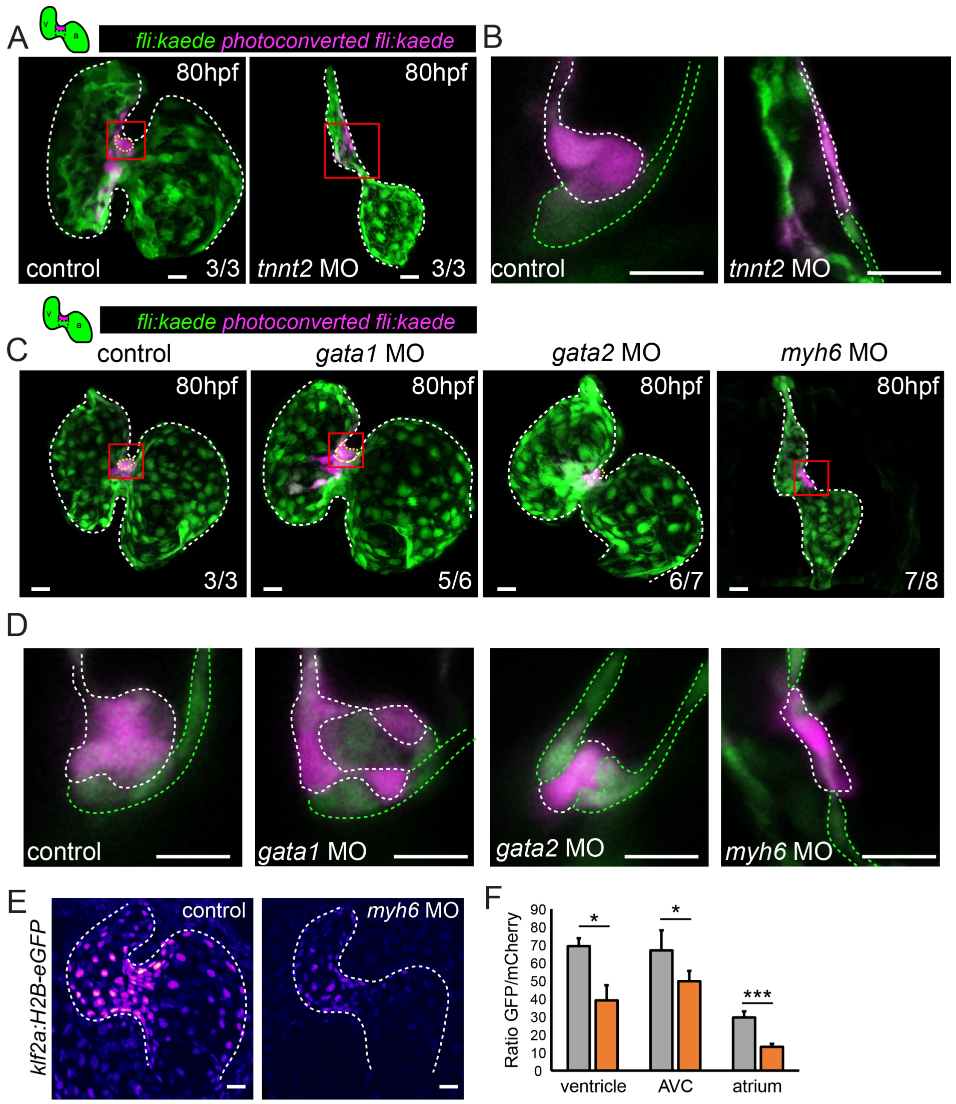

Supplementary Figure 3 – Defects in multi-layering occur in the presence of abnormal flow. Cells in the superior AVC of *Tg(fli:kaede)* embryos injected as shown were photoconverted in the superior AVC (schematic shown) at 48hpf and allowed to develop until 80hpf in all panels. (A) In *tnnt2a*MO-injected hearts, lacking heart contraction, multi-layering failed to occur by 80hpf (n=3 control, n=3 *tnnt2a*MO). Boxed red areas in (A) are shown in (B). Photoconverted cells remained in the single-layered endocardium. White dashed line highlights the photoconverted cells of the superior AVC. (C) Many cells were observed in the cardiac jelly of *gata1*MO-injected hearts (yellow dashed line highlights cells in the cardiac jelly) and intermingling of non-photoconverted (green) and photoconverted (magenta) cells was sometimes observed (n=3/6) (D). Boxed red areas from (C) are shown in (D). In contrast, multi-layering was impaired in *gata2*MO-injected and failed to occur in *myh6* MO-injected embryos. n=3 control, n=6 *gata1*MO, n=7 *gata2*MO, n=8 *myh6* MO. Green dashed line highlights the non-photoconverted cells of the superior AVC. (E and F) Quantification of GFP signal intensity in *klf2a:H2B-eGFP* embryos demonstrates reduced *klf2a* expression in *myh6* MO hearts (n=4 control hearts, n=3 *myh6*MO hearts). Error bar represents the standard deviation. Scale bars: 10 µm.

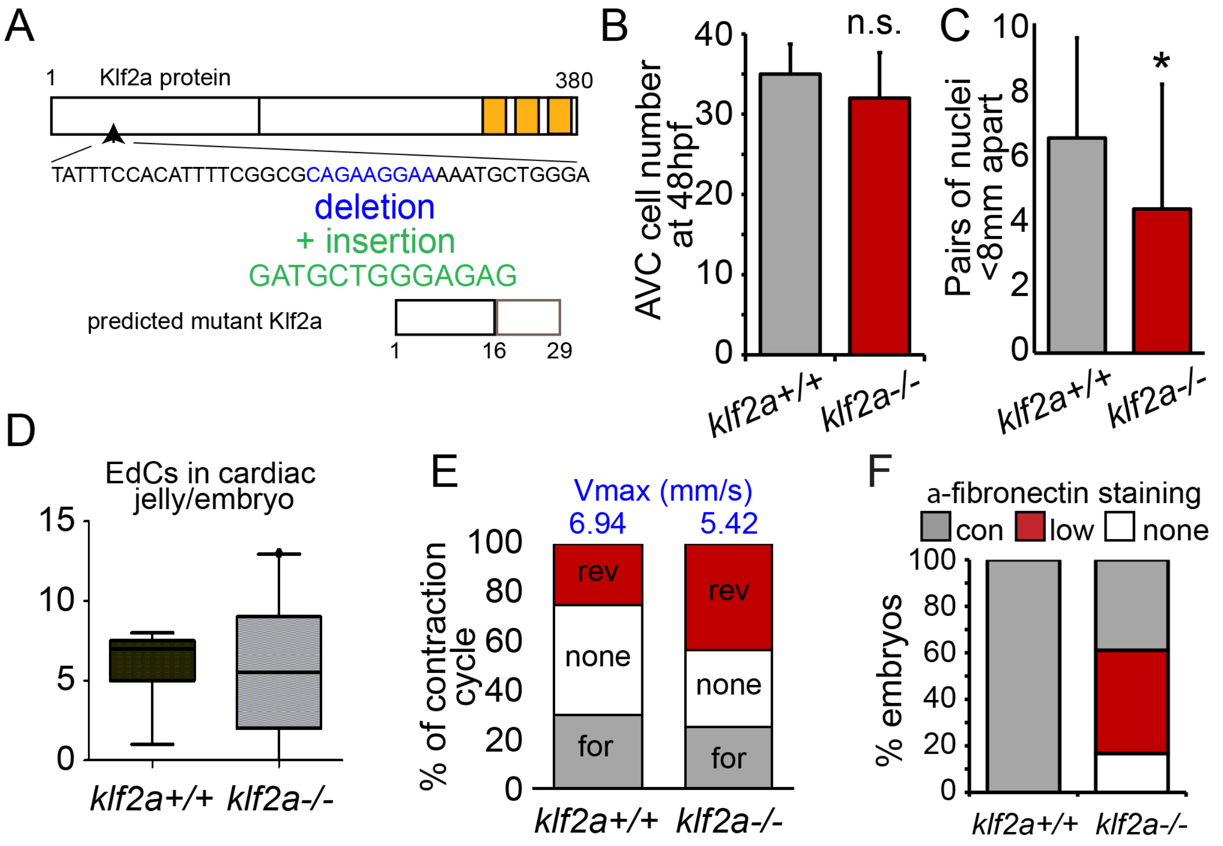

Supplementary Figure 4 – Characterisation of *klf2a*$^{-/-}$ mutants (A) Expanded nucleotide sequence shows the identity of the deletion (CAGAAGGAA) and concomitant insertion (GATGCTGGGAGAG) in the *klf2a* gene and its location in the Klf2a protein in our *klf2a* mutants. Computational translation predicts the translation of the first 16 amino acids of Klf2a, followed by an additional 13 amino acids and a premature stop codon. (B) Quantification of cell numbers in the AVC of *klf2a*$^{+/+}$ (n=11) and *klf2a*$^{-/-}$ (n=12) embryos at 48hpf showed no significant difference at the onset of valve formation. (C) Analysis of cell clustering showed fewer pairs of cells <8μm apart in *klf2a*$^{-/-}$ (n=12) compared to *klf2a*$^{+/+}$ controls (n=9). (D) Cell numbers in the cardiac jelly are altered in klf2a$^{-/-}$ mutants (n=8 klf2$^{+/+}$, n=13 *klf2a*$^{-/-}$*)*. Horizontal line shows the mean value, whiskers show the maximum and minimum values. (E) The flow profile was assessed by tracking red blood cells through the AVC in 3 consecutive heartbeats in *klf2a*$^{+/+}$ and *klf2a*$^{-/-}$ embryos. The average proportion of forward (for), reversing (rev) and no flow (none) within the AVC is shown (n=2 and 3 embryos, respectively). The average maximum velocity (Vmax) in mm/s is also shown (blue text). (F) Quantification of the % embryos displaying different levels of anti-Fibronectin staining in *klf2a*$^{-/-}$ mutants and controls. Error bar represents the standard deviation.

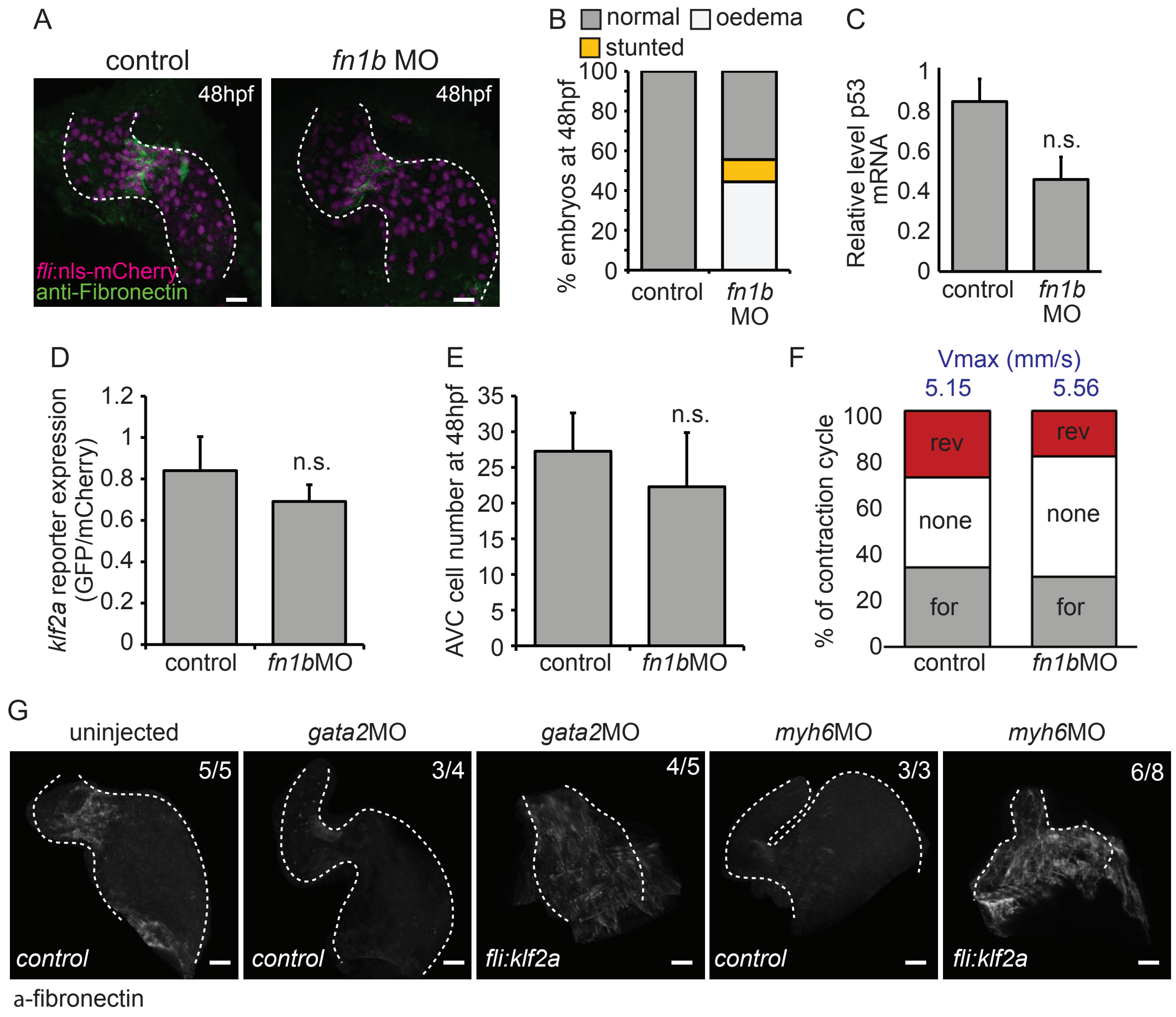

Supplementary Figure 5 – Characterisation of *fn1b*MO-injected embryos. (A) Immunofluorescence analysis shows reduced Fibronectin-positive staining (green) in the AVC of *fli:nlsmCherry* embryos injected with *fn1b*MO at 48 hpf (n=5 control, n=5 *fn1b*MO). (B) Quantification of overall morphological defects after *fn1b*MO injection (n=279 control, n=286 *fn1b*MO). (C) RT-qPCR analysis shows no significant increase in p53 expression following injection of *fn1b*MO compared to controls (RNA extracted from 3 pools of 20 embryos). (D) Relative *klf2a* expression in *klf2a:H2BEGFP* embryos and (E) cell numbers at 48hpf are not affected by injection of *fn1b*MO (n=4 control, n=7 *fn1b*MO). (F) The flow profile was assessed by tracking red blood cells through the AVC in 3 consecutive heartbeats in control and *fn1b*MO embryos. The average proportion of forward (for), reversing (rev) and no flow (none) within the AVC is shown (n=2 and 3 embryos, respectively). The average maximum velocity (Vmax) in mm/s is also shown (blue text). (G) *fli:kaede* (control) and *fli:klf2a* embryos injected with *gata2* and *myh6* morpholinos were immunostained with anti-fibronectin antibody. Fibronectin synthesis can be seen throughout the heart in *gata2* and *myh6* morphants overexpressing *klf2a*. Error bar represents the standard deviation. Scale bars: 10 μm.
